# FAP PET identifies earlycardiac molecular changesinduced by doxorubicin chemotherapy

**DOI:** 10.1172/jci.insight.191058

**Published:** 2025-10-23

**Authors:** Chul-Hee Lee, Onorina L. Manzo, Luisa Rubinelli, Sebastian E. Carrasco, Sungyun Cho, Thomas M. Jeitner, John Babich, Annarita Di Lorenzo, James M. Kelly

**Affiliations:** 1 Molecular Imaging Innovations Institute and Department of Radiology,; 2 Department of Pathology and Laboratory Medicine,; 3 Cardiovascular Research Institute,; 4 Brain and Mind Research Institute, and; 5 Laboratory of Comparative Pathology, Weill Cornell Medicine, New York, New York, USA.; 6 Laboratory of Comparative Pathology, Memorial-Sloan Kettering Cancer Center, New York, New York, USA.; 7 Laboratory of Comparative Pathology, Rockefeller University, New York, New York, USA.; 8 Laboratory of Comparative Pathology, Hospital for Special Surgery, New York, New York, USA.; 9 Department of Pharmacology,; 10 Sandra and Edward Meyer Cancer Center, and; 11 Citigroup Biomedical Imaging Center, Weill Cornell Medicine, New York, New York, USA.

**Keywords:** Cardiology, Therapeutics, Cardiovascular disease, Diagnostic imaging

## Abstract

Anthracycline chemotherapy, widely used in cancer treatment, poses a significant risk of cardiotoxicity that results in functional decline. Current diagnostic methods poorly predict cardiotoxicity because they do not detect early damage that precedes dysfunction. Positron emission tomography (PET) is well suited to address this need when coupled with suitable imaging biomarkers. We used PET to evaluate cardiac molecular changes in male C57BL/6J mice exposed to doxorubicin (DOX). These mice initially developed cardiac atrophy, experienced functional deficits within 10 weeks of treatment, and developed cardiac fibrosis by 16 weeks. Elevated cardiac uptake of [^68^Ga]Ga-FAPI-04, a PET tracer targeting fibroblast activation protein **α** (FAP), was evident by 2 weeks and preceded the onset of functional deficits. Cardiac PET signal correlated with FAP expression and activity as well as other canonical indicators of cardiac remodeling. By contrast, cardiac uptake of [^18^F]DPA-714 and [^18^F]MFBG, which target translocator protein 18 kDa and the norepinephrine transporter, respectively, did not differ between the DOX animals and their controls. These findings identify FAP as an early imaging biomarker for DOX-induced cardiac remodeling in males and support the use of FAP PET imaging to detect some cancer patients at risk for treatment-related myocardial damage before cardiac function declines.

## Abstract 2

Positron emission tomography using a probe that targets fibroblast activation protein detects incipient cardiotoxicity induced by doxorubicin before overt declines in heart function

## Graphical abstract

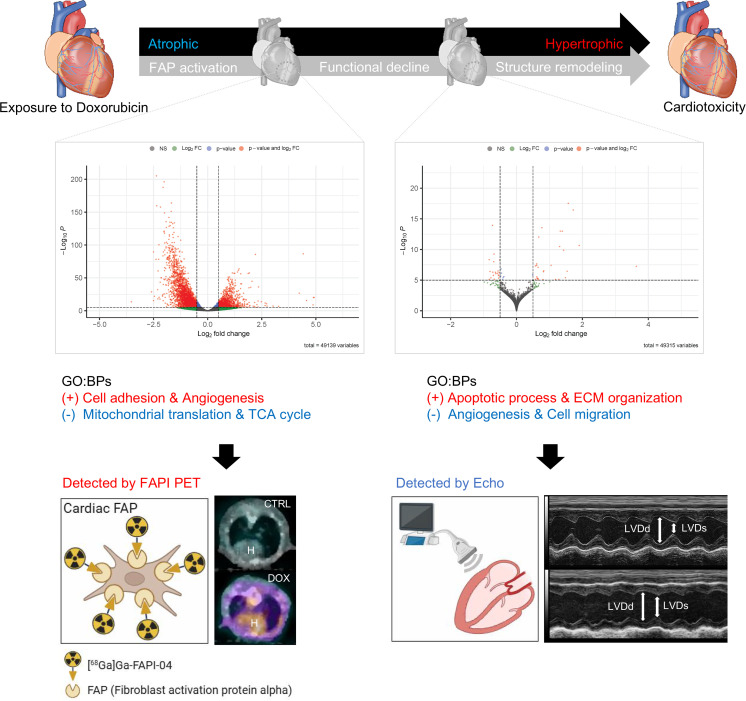

## Introduction

Doxorubicin (DOX), an anthracycline chemotherapeutic, is the drug of choice for treating a broad range of cancers that afflict adults and, especially, children (1, 2). For example, 60% of pediatric patients with cancer currently receive DOX as part of their cancer treatment (3). However, despite being a mainstay of anticancer therapy, DOX can induce cardiotoxicity, a spectrum of cardiac pathologies that can result in heart failure (4, 5). Indeed, up to 70% of the total adverse events reported from this drug relate to cardiac health (6, 7). Childhood cancer survivors are particularly susceptible to the long-term effects of DOX. More than 10% of pediatric patients treated with DOX develop cardiotoxicity and severe heart disease in adulthood (8, 9). We hypothesize that early diagnosis of cardiotoxicity due to DOX will lead to more effective interventions that improve the long-term outcomes of cancer treatment.

Assessment of left ventricular ejection fraction (LVEF) by echocardiography (echo) is currently the gold standard for evaluating cardiac function in patients with suspected cardiotoxicity (10). Despite the introduction of speckle tracking-based deformation analysis to detect subclinical cardiotoxicity (11, 12), the technique remains a measure of overall function rather than the damage to cardiomyocytes that triggers a cascade of events that compromises cardiac performance (13, 14). Blood biomarkers of cardiac injury, such as circulating cardiac troponins and B-type natriuretic peptides, are also insufficiently sensitive to detect cardiotoxicity arising from DOX treatment (15). To detect incipient cardiotoxicity arising either during or after chemotherapy therefore requires the validation of new biomarkers related to specific disease-causing pathophysiologies.

Given the limitations of current diagnostic methods, we sought to evaluate positron emission tomography (PET) for detecting incipient cardiotoxicity. We hypothesized that imaging 3 processes proposed to be induced by anthracyclines — cardiac tissue remodeling (16), inflammation (17), and excessive cardiac sympathetic activation (18) — with suitable PET radioligands would result in the early detection of DOX-induced cardiac injury. These probes, which target fibroblast activation protein α (FAP) (19–22), translocator protein 18 kDa (TSPO) (23–25), and the norepinephrine transporter (NET) (26, 27), respectively, were developed for applications in oncology and neurodegenerative diseases but as of yet have not been evaluated in the context of cardiotoxicity. Here, we employ a preclinical model of DOX-induced cardiotoxicity to demonstrate that PET imaging of FAP successfully detects the early response of the male heart to DOX exposure that precedes the emergence of cardiac dysfunction. We provide additional evidence that DOX exposure induces FAP in cardiomyocytes as part of the dynamic tissue remodeling process initiated by damage to these cells. These findings support the use of FAP PET to monitor molecular changes in hearts exposed to anthracycline chemotherapeutics.

## Results

### *Exposure to DOX initially induces cardiac atrophy in male mice that ultimately results in left ventricular dysfunction*.

**Figure 1:**
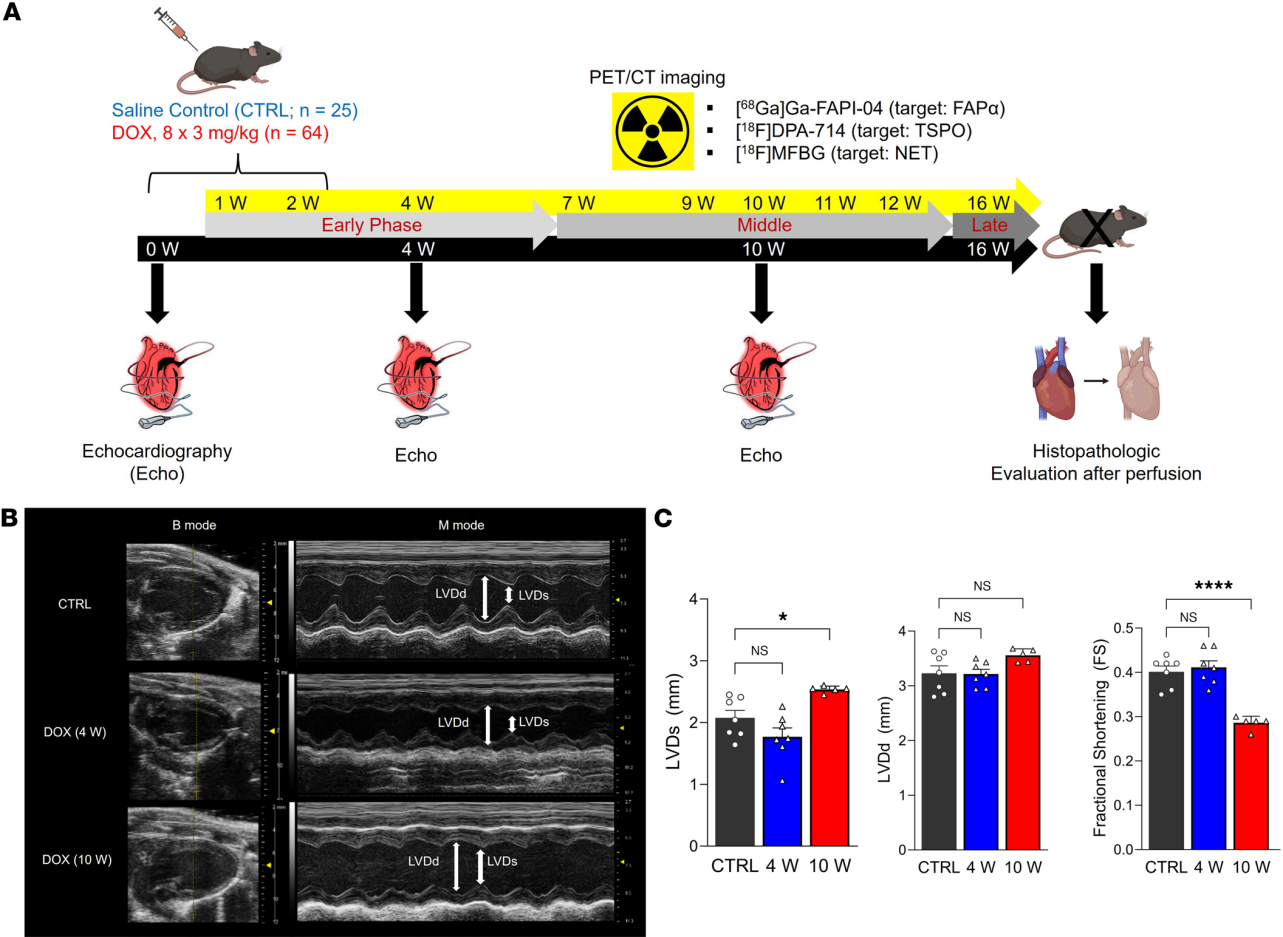
Systemic DOX administration induces cardiac functional decline. (**A**) Experimental design for in vivo and ex vivo studies. Illustrations were created with BioRender.com. (**B**) Representative echo imaging of control and DOX groups at 4 and 10 weeks. (**C**) Functional parameters were determined from the echo scans at 4 and 10 weeks (control, *n* = 7; 4 weeks, *n* = 6; and 10 weeks, *n* = 5). FS was calculated from LVDd and LVDs. Statistical comparisons were performed using a 1-way ANOVA with multiple comparisons. *P < 0.05; ****P < 0.0001; DOX, doxorubicin; echo, echocardiogram; FS, fractional shortening; LVDd, left ventricular end-diastolic diameter; LVDs, left ventricular end-systolic diameter.

Administration of intraperitoneal DOX (cumulative dose 24 mg/kg) over 2 weeks to male C57BL/6J mice induced substantial changes in the hearts of these mice compared with the control cohort. We assessed these changes over 16 weeks by serial echo and microPET/CT imaging and corresponding ex vivo tissue analysis (Figure 1A). Echo imaging highlighted compromised cardiac function at 10 weeks but not at 4 weeks (Figure 1B), as evidenced by a 20% increase in left ventricle end-systolic diameter (LVDs) and a statistically significant decrease in fractional shortening to less than 30% in the DOX group (Figure 1C). Left ventricle end-diastolic diameter (LVDd) did not significantly change over time. The heart rates of the mice in the control and DOX groups were largely unchanged (Supplemental Figure 1A).

**Figure 2:**
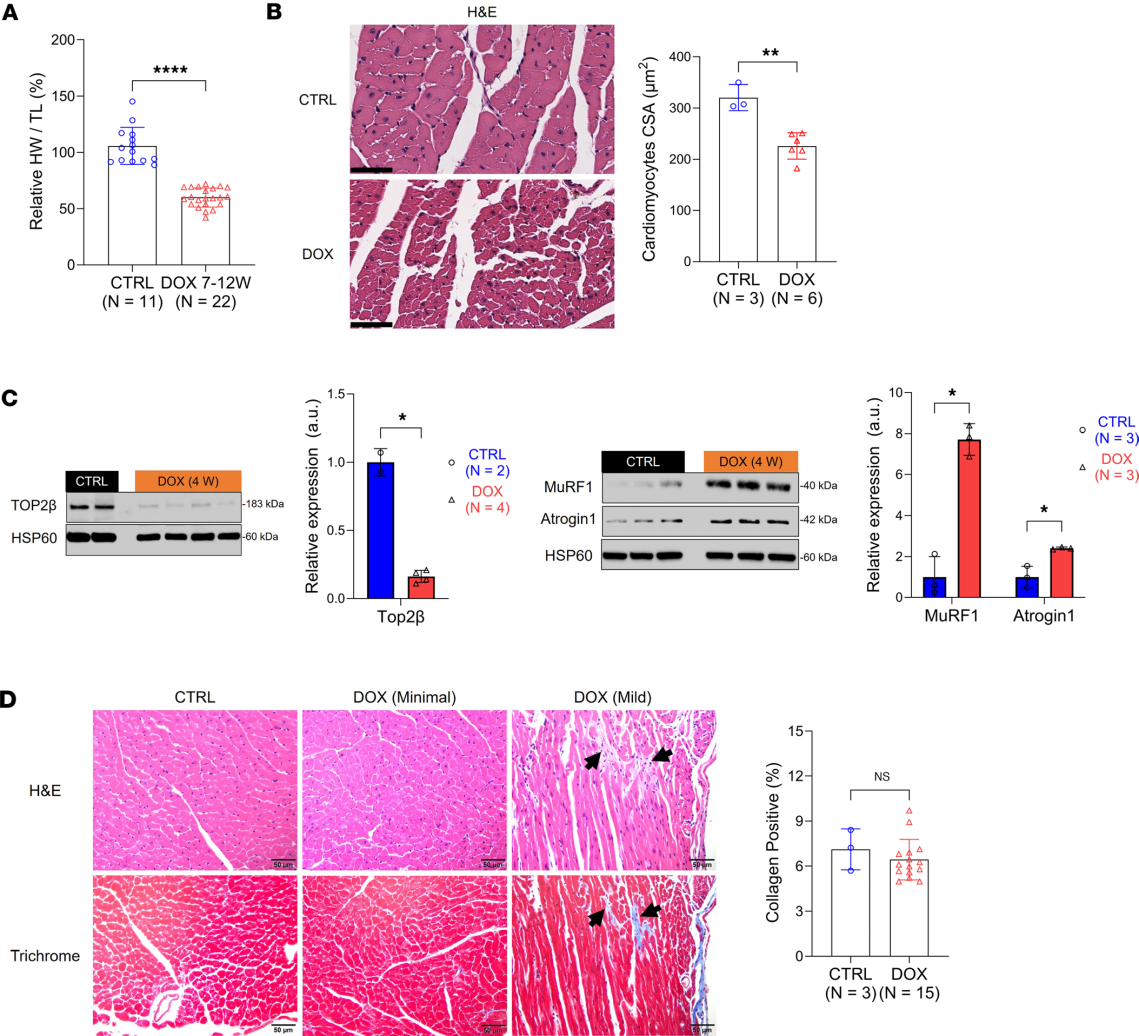
Systemic DOX administration to male mice induces cardiomyocyte atrophy that precedes the onset of fibrosis. (**A**) HW/TL ratio. (**B**) Assessment of cardiomyocyte CSA from tissue slices stained with H&E. Scale bar: 50 μm. (**C**) Western blot analysis of cardiac Atrogin1, MuRF1, and TOP2β from heart samples collected 4 weeks after initial DOX exposure (*n* = 2–4 per group). HSP60 is used as a reference. Region of interest (ROI) quantification was performed using ImageJ (NIH). (**D**) Representative H&E and trichrome stains from tissues collected at 10 weeks. Tissue collected from the DOX group was designated “minimal” or “mild” based on trichrome positive staining. Mild myocardial fibrosis was occasionally and regionally detected in DOX-treated hearts (black arrows). Positive regions were defined as the deposition of collagen fibrils between cardiomyocytes. Scale bar: 50 μm. Data are presented as the mean ± SD. Statistical comparisons were performed using a Mann-Whitney test (**A**) or an unpaired 2-tailed *t* test (**B**–**D**). *P < 0.05; **P < 0.01; ****P < 0.0001; HW/TL, heart weight-to-tibia length; CSA, cross-sectional area; H&E, hematoxylin and eosin; HSP60, heat shock protein 60; a.u., arbitrary unit.

Next, we examined physiological changes in DOX-treated male mice compared with controls, focusing on a time interval (7 to 12 weeks) in which cardiac dysfunction emerged, which we defined as the “middle phase” of pathology (Figure 1A). In agreement with multiple reports (28, 29), body weights were lower in the DOX-treated mice (Supplemental Table 1), and the heart weight (HW) to tibia length (TL) ratio was 40% lower in these mice (Figure 2A and Supplemental Table 2). There were no significant differences in TL between the 2 groups (Supplemental Figure 1B), which indicates the onset of cardiac atrophy as a result of DOX exposure. By H&E staining and morphometric analysis (Supplemental Table 3), we determined that cardiac atrophy was due to significantly decreased cardiomyocyte size in the DOX-treated hearts compared with the controls (Figure 2B). This finding was reinforced by increased cardiac expression of atrogin1 and MuRF1, 2 established markers of cardiomyocyte atrophy (28). Expression of these markers increased notably during the early phase of pathology (weeks 1–7) at both the gene and protein levels following initial DOX exposure, with notable increases in expression at 4 weeks (Figure 2C and Supplemental Figure 1, C and D). While atrogin1 gene expression remained elevated throughout the middle phase, *Murf1* gene expression decreased to baseline at the 10-week time point. Conversely, the expression of topoisomerase-2β (TOP2β), a known target of DOX and primary contributor to cardiotoxicity (29), was significantly reduced at the 4-week time point (Figure 2C).

As evidence that cardiac atrophy was not due to loss of cardiomyocytes, we examined cardiac tissue histologically. To our surprise, in light of multiple literature reports of DOX-induced cardiac fibrosis in mice (30, 31), cardiomyocytes in the DOX-treated mouse hearts (*n* = 15) collected in weeks 7–12 showed little evidence of histopathological change, consisting of only a mild degree of individual cardiomyocyte necrosis and degeneration and/or focal to multifocal areas of myocardial fibrosis in a small cohort (*n* = 4 out of 15, Supplemental Figure 1E). In agreement with these findings, there was no difference in trichrome staining between the groups at this time point (Figure 2D and Supplemental Table 3). Collectively, and in agreement with literature reports (32), these results confirm that functional deficits follow reductions in cardiomyocyte volume (without cardiomyocyte loss) but precede overt myocardial fibrosis in our DOX model.

### *[^^68^^Ga]Ga-FAPI-04 PET detects early increases in cardiac tissue FAP expression and activity in male mice*.

**Figure 3:**
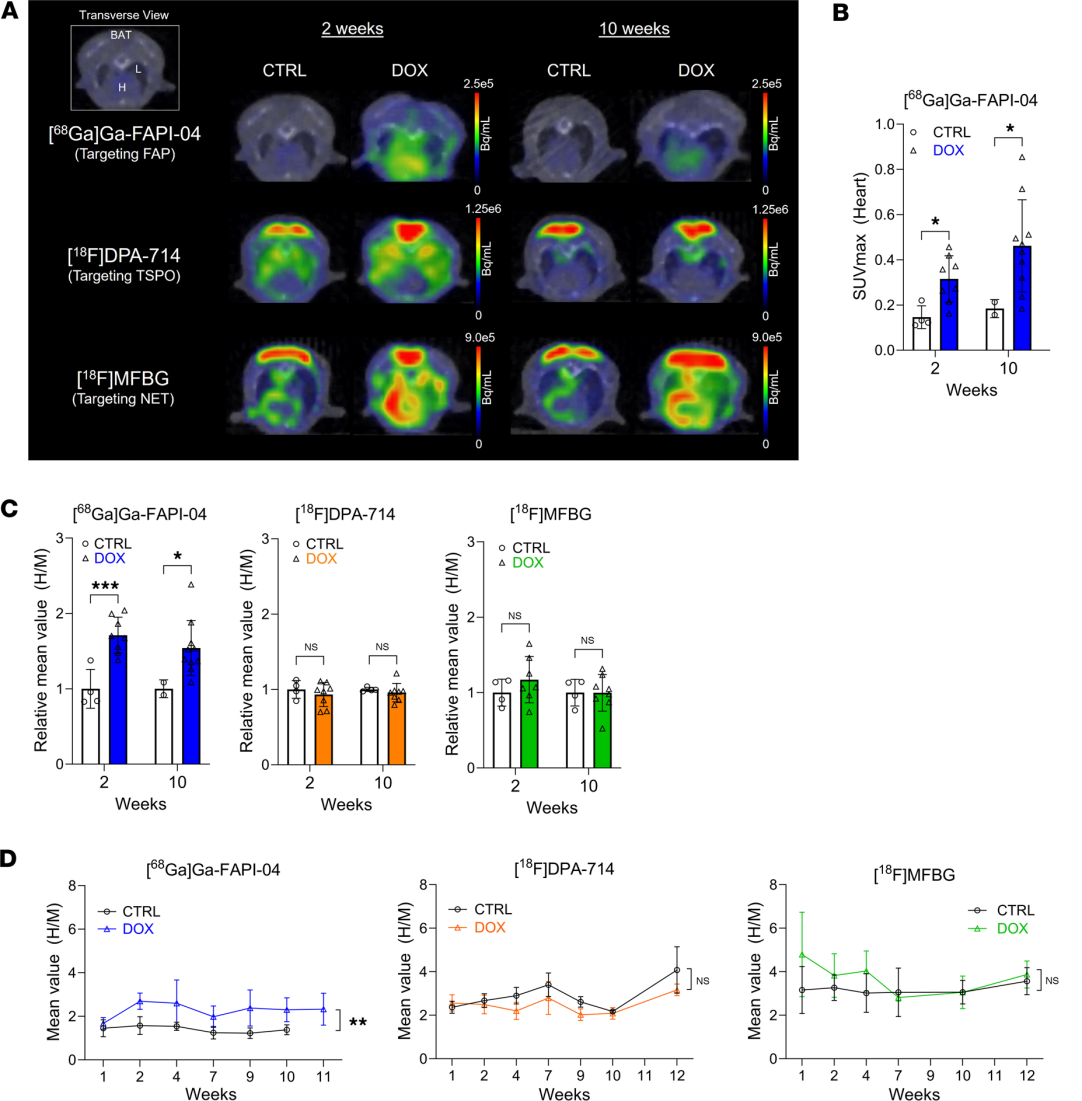
Early-stage FAP expression is detectable with [^68^Ga]Ga-FAPI-04 PET. (**A**) Representative summed PET/CT fusion transaxial MIP images at 2 and 10 weeks after initial DOX exposure. Mice were imaged with [^68^Ga]Ga-FAPI-04 (top), [^18^F]DPA-714 (middle), or [^18^F]MFBG (bottom). Images were acquired 60 minutes postinjection. BAT, brown adipose tissue; H, heart; L, lung. (**B**) SUVmax of [^68^Ga]Ga-FAPI-04 in cardiac tissues at 2 and 10 weeks. (**C**) Quantitation of cardiac PET signals normalized to skeletal (calf) muscle uptake (heart/muscle ratio, H/M) at 2 and 10 weeks. (**D**) Comparison of image-derived cardiac uptake of [^68^Ga]Ga-FAPI-04, [^18^F]DPA-714, and [^18^F]MFBG over the time course of the experiment. Quantification of cardiac PET uptake of **C** and **D** was performed using AMIDE and normalized to skeletal (calf) muscle uptake. At least 3 mice were imaged at each time point, with the specific number reported in Supplemental Table 4. Data are presented as the mean ± SD. Statistical comparisons were performed using an unpaired 2-tailed *t* test, with Welch’s correction in **D**. *P < 0.05; **P < 0.01; ***P < 0.001. FAP, fibroblast activation protein α; NET, norepinephrine transporter; TSPO, translocator protein 18 kDa; SUVmax, maximum standardized uptake value; MIP, maximum intensity projection.

To test our hypothesis that PET imaging detects cardiotoxic effects of DOX before functional impairment arises, we conducted serial microPET/CT imaging in male mice over 12 weeks using [^^68^^Ga]Ga-FAPI-04, [^^18^^F]DPA-714, and [^^18^^F]MFBG (Figure 3A and Supplemental Table 4). We determined standardized uptake values (SUVs) to compare cardiac uptake between groups. Using this metric, we observed significantly increased cardiac [^^68^^Ga]Ga-FAPI-04 SUVmax in the mice exposed to DOX at 2 weeks, at which point there is no evidence of cardiac functional decline, and at 10 weeks, when dysfunction has emerged (Figure 3B). Additionally, as the background signal was consistently higher in the mice exposed to DOX, we normalized cardiac uptake to skeletal muscle (heart to muscle, H/M). Skeletal muscle is also subject to DOX-induced toxicity (28) and therefore controls for systemic effects. The volume of interest over the calf muscle measured approximately 25 mm^^3^^ and did not vary between animals and groups (Supplemental Figure 2A). Although [^^68^^Ga]Ga-FAPI-04 SUVmax in muscle was slightly higher at 10 weeks than at 2 weeks, the difference was not significant (Supplemental Figure 2B). In agreement with the SUVmax comparison, the [^^68^^Ga]Ga-FAPI-04 H/M ratio in the DOX group was significantly elevated, increasing by 1.7-fold over the control group at 2 weeks (*P* < 0.001) and remaining at least 1.5-fold higher through 10 weeks (*P* = 0.040; Figure 3, A and C; and Supplemental Table 5). By contrast, there was no significant difference in the H/M ratio of [^^18^^F]DPA-714 and [^^18^^F]MFBG between the control and DOX groups at 2 weeks or 10 weeks. Moreover, serial PET imaging conducted over 12 weeks using all 3 probes demonstrated statistically significant differences in H/M ratio exclusively with [^^68^^Ga]Ga-FAPI-04 (Figure 3D). This was also the case when imaging time points were grouped into early (1–7 weeks) and middle (7–12 weeks) disease phases (Supplemental Figure 2C). These results support cardiac FAP as an imaging biomarker for detecting early-stage cardiotoxicity induced by anthracycline in males.

**Figure 4:**
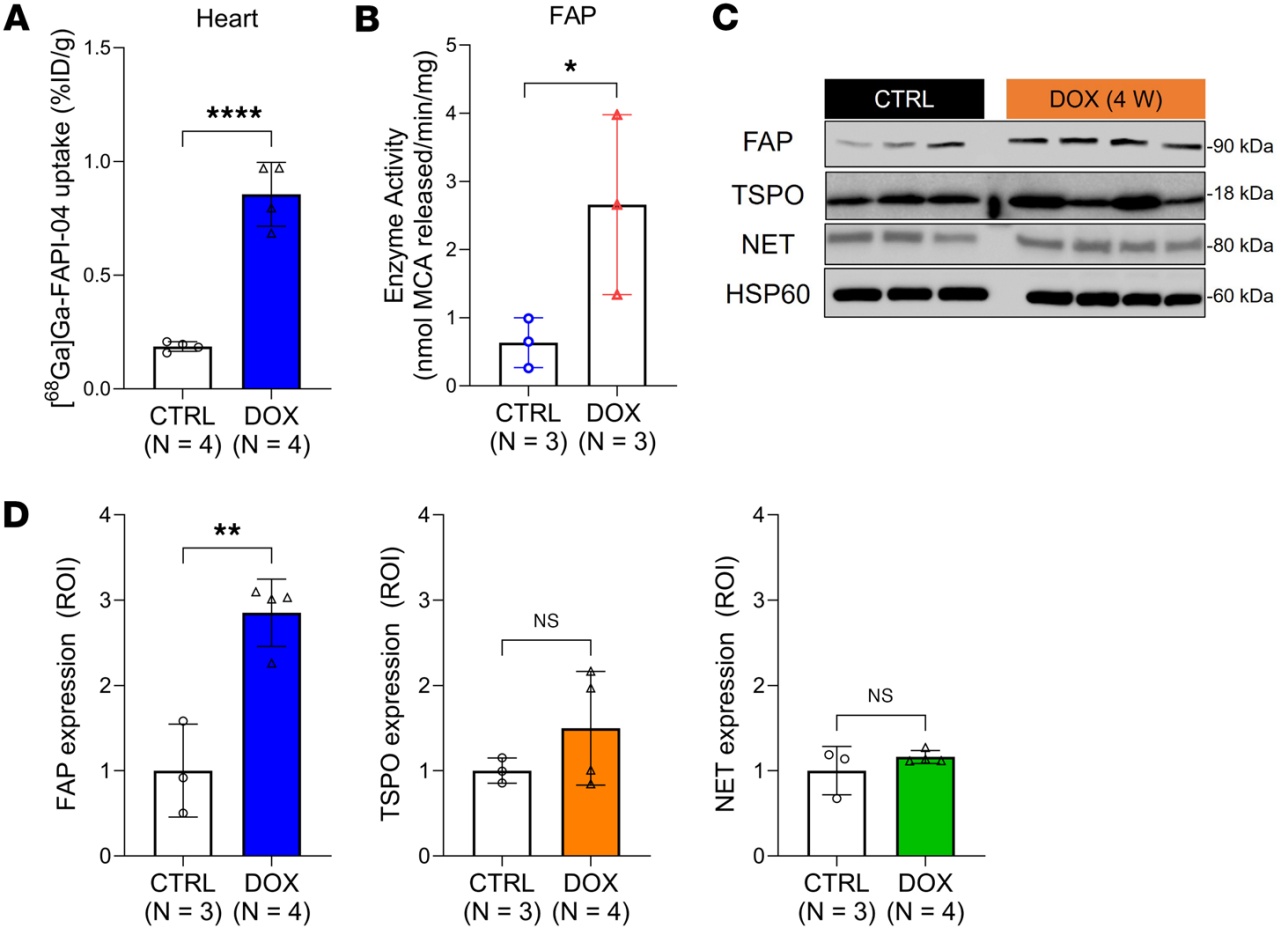
Cardiac [^68^Ga]Ga-FAPI-04 PET corresponds with tissue levels of FAP expression and activity. (**A**) Cardiac uptake of [^68^Ga]Ga-FAPI-04 determined by ex vivo biodistribution at 4 weeks. Hearts were exsanguinated and patted dry before tissue counting. (**B**) FAP enzyme activity in heart tissue extracts taken at 4 weeks. (**C**) Western blot analysis of cardiac FAP, TSPO, and NET expression. HSP60 is used as a reference. (**D**) ROI quantification of each protein level was performed using ImageJ. Data are presented as the mean ± SD. Statistical comparisons were performed using an unpaired 2-tailed (**A** and **D**) or 1-tailed (**B**) *t* test. *P < 0.05; **P < 0.01; ****P < 0.0001. FAP, fibroblast activation protein α; MCA, 7-methoxycoumarin-4-acetic acid; NET, norepinephrine transporter; TSPO, translocator protein 18 kDa; HSP60, heat shock protein 60; ROI, region of interest; ID, injected dose.

Next, we correlated the cardiac uptake of the PET agents with protein and mRNA expression in the hearts of male mice. Cardiac tissues were perfused and excised for ex vivo analysis at 4 weeks, at which time no functional deficits were evident by echo imaging. Consistent with our imaging results, we confirmed by γ-counting that cardiac [^^68^^Ga]Ga-FAPI-04 uptake was significantly higher in the DOX-treated mice (Figure 4A) and remained significantly (1.5-fold) higher when normalized to excised muscle tissue (Supplemental Figure 2D). In contrast, consistent with multiple clinical reports (33, 34), plasma FAP levels were 3.2-fold higher in control mice compared with the DOX group (Supplemental Figure 2E). These findings confirm that the higher cardiac SUV observed in our PET images is not due to increased circulating FAP activity in the mice exposed to DOX.

We also observed a 4.2-fold increase in cardiac FAP activity in tissue samples taken from the DOX group (Figure 4B). In parallel, we found a 2.9-fold increase in cardiac expression of FAP protein upon DOX treatment but no change in TSPO or NET expression (Figure 4, C and D). Similarly, although we observed greater heterogeneity in the tissues exposed to DOX, *Fap* mRNA expression, analyzed using 3 different primers (Supplemental Table 6), was significantly elevated (*P* < 0.0001) in these hearts compared with controls (Supplemental Figure 2F). *Tspo* mRNA levels did not differ (Supplemental Figure 2G). *Fap* gene and FAP protein expression levels were elevated in the mice treated with DOX at weeks 2, 7, and 10 (Supplemental Figure 2, H and I) and directly related to cardiac [^^68^^Ga]Ga-FAPI-04 SUV (Supplemental Figure 2J).

### *Increased cardiac [^^68^^Ga]Ga-FAPI-04 uptake is significantly correlated to FAP expression in cardiomyocytes*.

**Figure 5:**
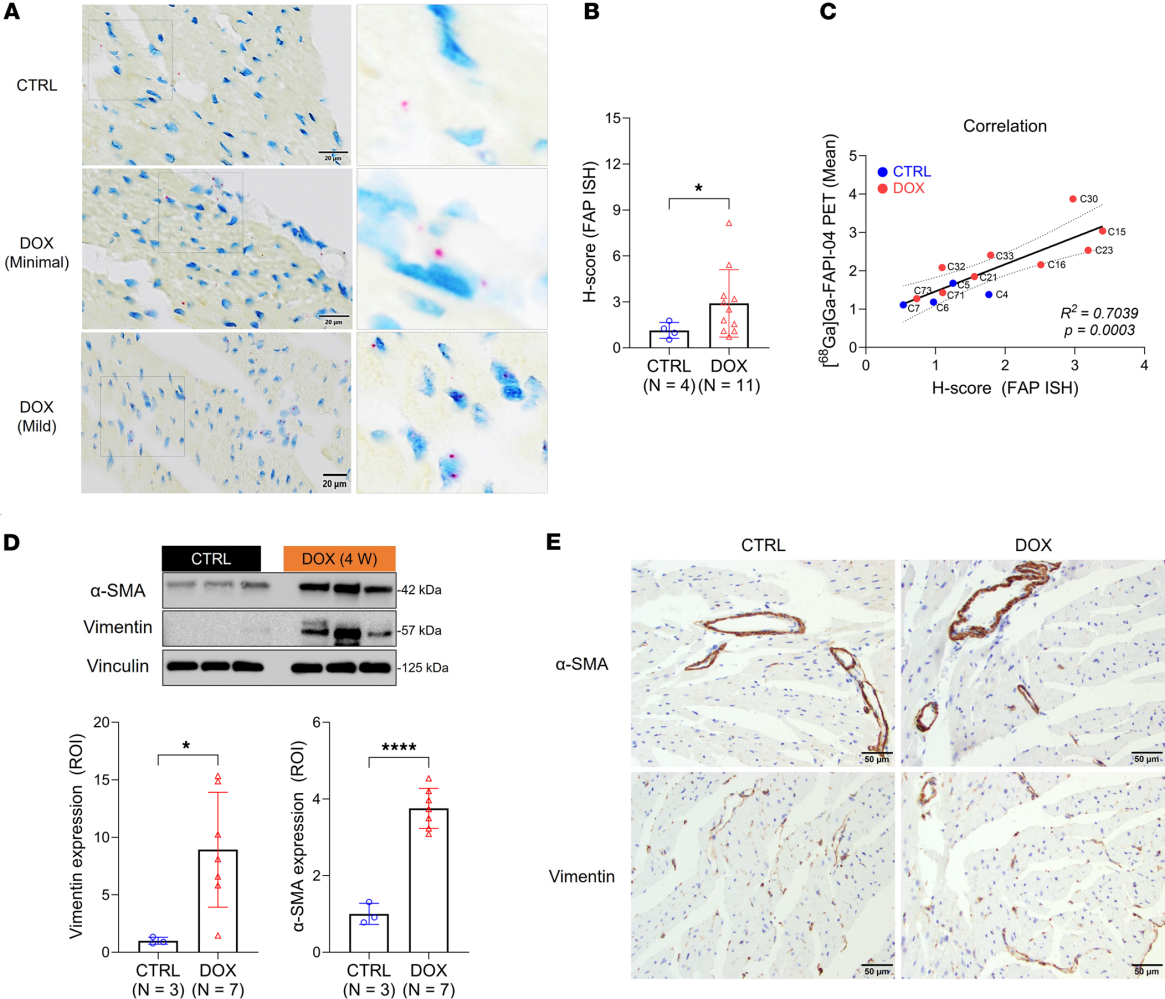
Cardiac [^68^Ga]Ga-FAPI-04 uptake correlates with FAP expression in cardiomyocytes in mouse tissue. (**A**) Representative FAP stains. Left: *Fap* mRNA (punctate red dots) was detected in the cytoplasm and/or nuclei of cardiomyocytes and/or epicardial stromal cells (insets). Scale bar: 20 μm. Right: A quadrupled image of the marked area. (**B**) Comparison of the H-score from *Fap* ISH staining between DOX and control groups. Quantitation was performed using QuPath software. (**C**) The correlation between [^68^Ga]Ga-FAPI-04 cardiac PET signals and H-score in the corresponding tissues (*P* = 0.0003). (**D**) Western blot analysis of cardiac α-SMA and Vimentin expression. Vinculin is used as a reference. ROI quantification of α-SMA and Vimentin protein levels. (**E**) Representative α-SMA and Vimentin immunostaining in cardiac tissue from control and DOX-treated mice. α-SMA is predominantly expressed in the cytoplasm of smooth muscle cells within cardiac arteries and arterioles. Vimentin immunolabeling is detected in a heterogeneous population of cardiac cells, including pericardial cells, pericytes, endothelial cells, and individual resident macrophages and/or stromal cells. Scale bar: 50 μm. All ROI quantification was performed using ImageJ. Data are presented as the mean ± SD. Statistical comparisons were performed using an unpaired 2-tailed *t* test (**B** and **D**) or a Pearson correlation test (**C**). *P < 0.05; ****P < 0.0001. ISH, in situ hybridization; α-SMA, α-smooth muscle actin; IHC, immunohistochemistry; ROI, region of interest.

Given that we observed increased cardiac FAP expression long before myocardial fibrosis, we sought to identify the cells responsible for these changes in expression. As our attempts to detect murine FAP by immunohistochemistry using commercially available antibodies were unsuccessful, we utilized in situ hybridization (ISH) to evaluate the spatiotemporal distribution of *Fap* nucleic acid in cardiac tissue (35). Contrary to our expectations, *Fap* nucleic acid was often detected in cardiomyocytes (Figure 5A). The H-score, an index of signal intensity, was increased in the samples taken from the DOX group and correlated linearly with cardiac [^^68^^Ga]Ga-FAPI-04 PET signal (adjusted *R*^^2^^ = 0.704) (Figure 5, B and C, and Supplemental Table 3). By contrast, and in agreement with our PET imaging findings, there was no significance in TSPO staining by immunohistochemistry (Supplemental Figure 3, A and B). None of the commercially available antibodies adequately stained murine NET, and we did not elect to pursue ISH for this target.

Although we did observe significantly increased expression of 2 markers of cardiac fibroblasts, α–smooth muscle actin (α-SMA) and vimentin, in the tissues exposed to DOX compared with controls by Western blot (Figure 5D), comparison of cardiac sections stained by immunohistochemistry did not identify differences in the fibroblast populations (Figure 5E). Rather, increased α-SMA and vimentin expression appears to reflect an increased population of smooth muscle cells, endothelial cells, and stromal cells in the hearts exposed to DOX.

**Figure 6:**
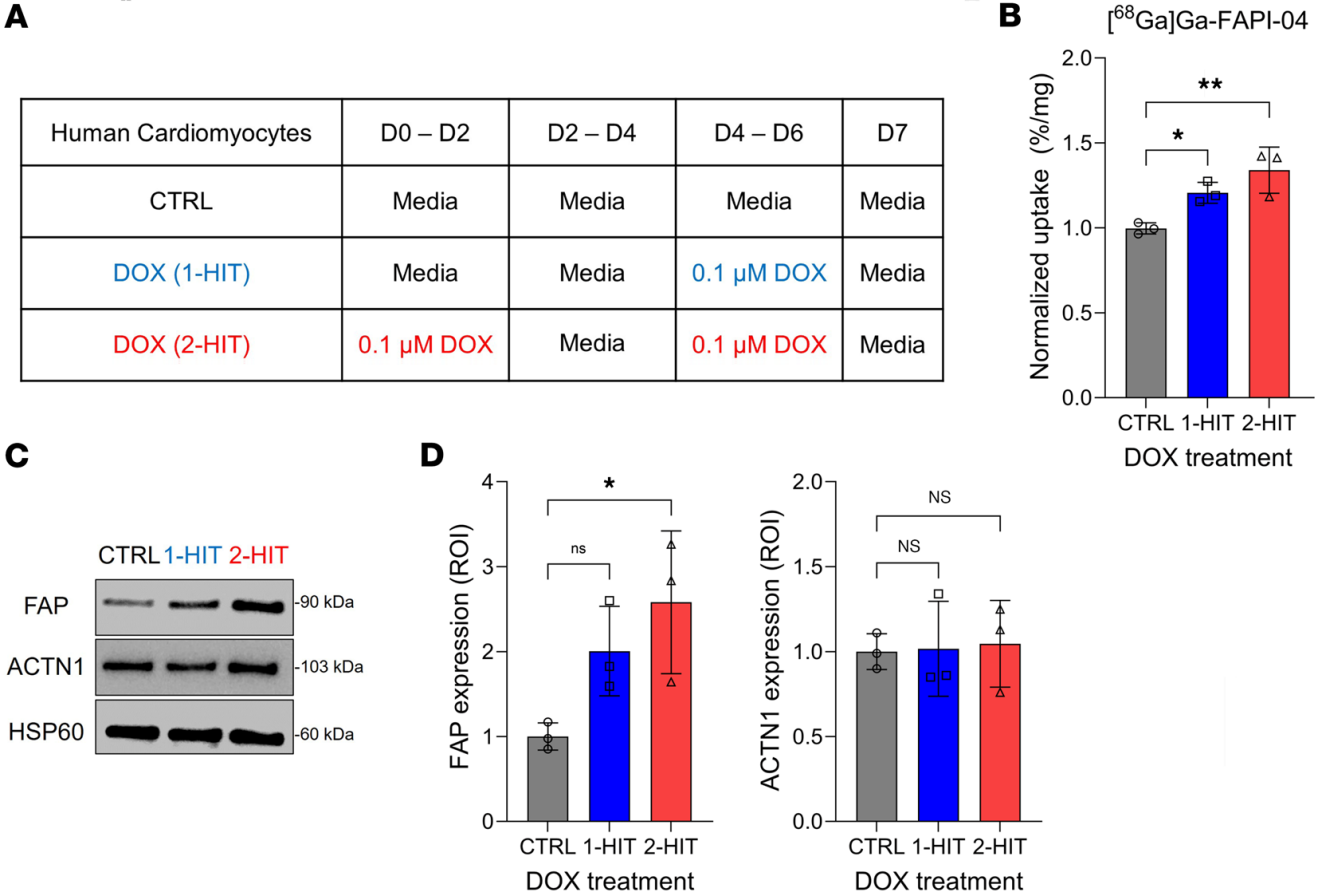
Human cardiomyocytes exposed to DOX increase [^68^Ga]Ga-FAPI-04 uptake. (**A**) Study plan: Human cardiomyocytes were plated and treated with vehicle (control, CTRL), 0.1 μM DOX for 48 hours starting on day 4 (1-hit), or 0.1 μM DOX for 2 × 48 hours (2-hit) starting on days 0 and 4 (*n* = 3/condition). (**B**) Uptake of [^68^Ga]Ga-FAPI-04 in human cardiomyocytes treated with vehicle, DOX (1-hit), or DOX (2-hit). (**C**) Western blot analysis of FAP and α–actinin-1 (ACTN1) expression in human cardiomyocytes extracted in the same condition. HSP60 is used as a reference. (**D**) ROI quantification of FAP and ACTN1 expression. All ROI quantification was performed using ImageJ. Data are presented as the mean ± SD. Statistical comparisons were performed using a 1-way ANOVA with multiple comparisons (**B** and **D**). *P < 0.05; **P < 0.01. FAP, fibroblast activation protein α; HSP60, heat shock protein 60; ROI, region of interest.

As FAP expression in cardiomyocytes has not been previously reported, though a recent study suggested that it might be induced by inflammation (36), we validated our histological observations in cultured human cardiomyocytes (HCMs) treated with 0.1 μM DOX for either 48 hours (1-hit) or 2 × 48 hours (2-hit) (37) (Figure 6A). The binding of [^^68^^Ga]Ga-FAPI-04 to these cells increased as a function of DOX exposure, leading to significantly higher uptake in the 2-hit cells than in untreated controls (Figure 6B). FAP protein expression in HCMs increased as a function of DOX exposure time (Figure 6, C and D). By contrast, the expression of ACTN1, a marker of contracting cardiomyocytes in culture (38), was unchanged, which suggests that DOX exposure does not change the fundamental nature of these cells. These results reveal that cardiac [^^68^^Ga]Ga-FAPI-04 PET signal correlates with FAP expression in cardiomyocytes.

### *DOX induces the early activation of FAP and other genes related to cardiac remodeling and mitochondrial dysfunction in male mice*.

**Figure 7:**
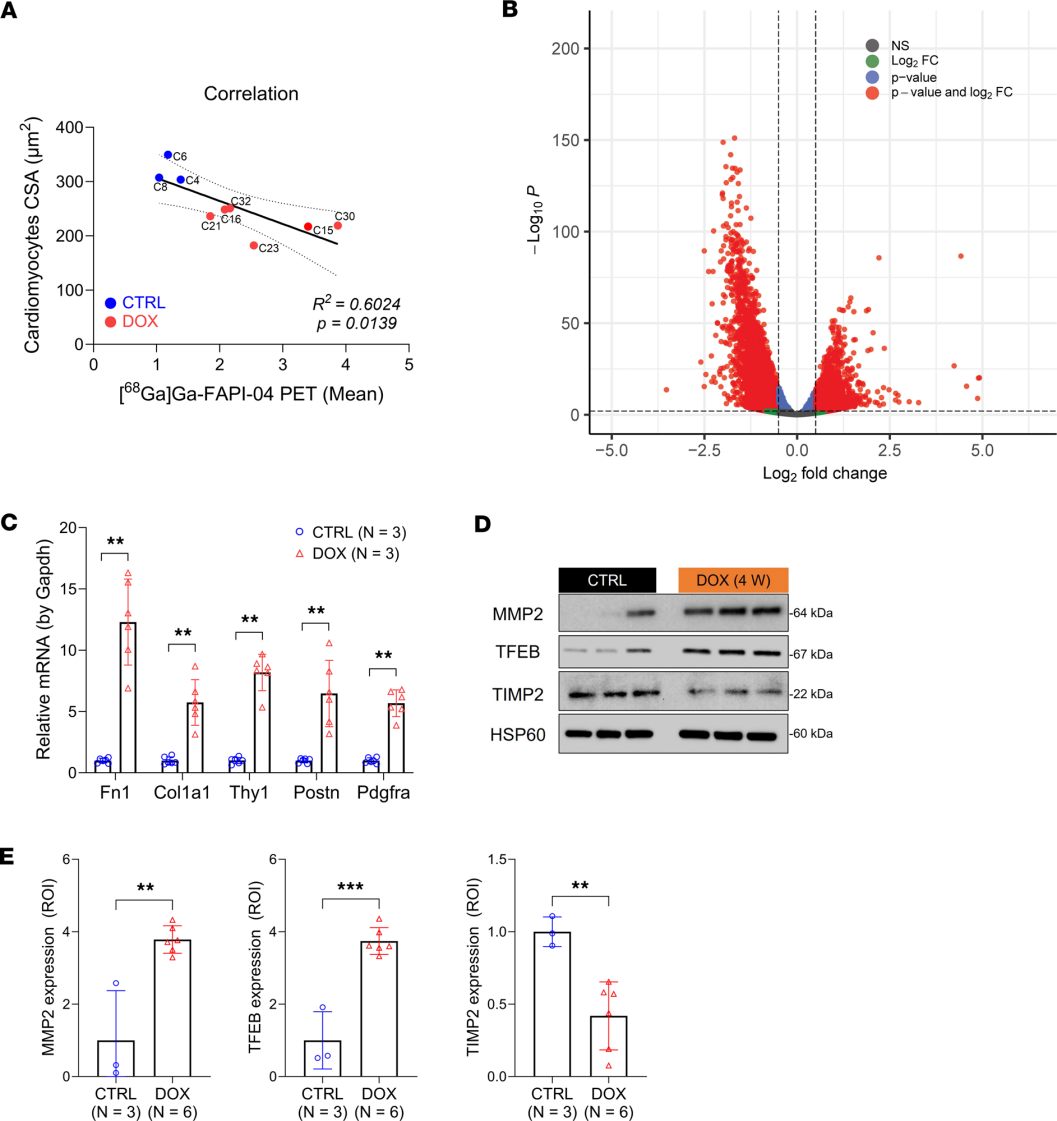
DOX triggers early activation of Fap and other genes linked to cardiac remodeling and mitochondrial dysfunction. (**A**) Correlation between cardiomyocyte CSA and [^68^Ga]Ga-FAPI-04 PET signal from cardiac tissues (*P* = 0.0139). (**B**) EnhancedVolcano plot from the bulk RNA sequencing at 4 weeks. A total of 49,139 variables were displayed (log2 fold change [FC] > |0.5|, *P* < 0.01). (**C**) RT-qPCR quantification of cardiac *Fn1*, *Col1a1*, *Thy1*, *Postn*, and *Pdgfra* mRNA expression using *Gapdh* as reference gene (*n* = 3 mice/group). Two different primers of the target gene were used to minimize the variation. (**D**) Western blot analysis of cardiac MMP2, TFEB, and TIMP2. (**E**) ROI quantification of MMP2, TFEB, and TIMP2 protein levels. HSP60 is used as a reference. All quantification was performed using ImageJ. Data are presented as the mean ± SD. Statistical comparisons were performed using a Pearson correlation test (**A**), a 2-way ANOVA (**C**), or an unpaired 2-tailed *t* test (**E**). **P < 0.01; ***P < 0.001. MMP2, matrix metalloproteinase-2; TFEB, transcription factor EB; TIMP2, tissue inhibitor of metalloproteinases 2; ROI, region of interest.

Cardiac [^^68^^Ga]Ga-FAPI-04 PET signal negatively correlated with cardiomyocyte size and HW/TL ratio (Figure 7A and Supplemental Figure 4A), which suggested to us that FAP contributed to compensatory remodeling of the extracellular matrix (ECM) necessitated by cardiomyocyte atrophy. To explore this hypothesis further, we conducted bulk RNA-sequencing analysis at 4 weeks. A total of 49,139 variables were analyzed to generate EnhancedVolcano plots based on differentially expressed genes (DEGs) (log2 fold-change [FC] > |0.5|, *P* < 0.001) (Figure 7B). Although neither the *Fap*, *Tspo*, nor *Slc6a2* (NET) genes were significantly altered in the overall population of DEGs, *Fap* gene expression was significantly elevated (*P* = 0.013) in DOX-exposed mice when normalized by fragments per kilobase of transcript per million (Supplemental Figure 4, B and C).

Next, we identified DEGs with clear distinctions between the DOX and control groups (*P* < 0.01, log2FC ≥ 0.55 or log2FC ≤ –0.85) to perform Gene Ontology (GO) and Kyoto Encyclopedia of Genes and Genomes (KEGG) pathway enrichment using the Database for Annotation, Visualization and Integrated Discovery (DAVID) and STRING databases. Numerous biological processes (BPs) related to cell and tissue remodeling were highlighted among the most significantly upregulated pathways (Supplemental Figure 4, D and E, and Supplemental Tables 7 and 8). By contrast, as previously reported (39), many GO:BPs associated with mitochondrial function were significantly downregulated (Supplemental Figure 4F and Supplemental Table 9). These results suggest that DOX exposure rapidly triggers the activation of genes involved in cardiac tissue remodeling and the inactivation of genes that contribute to mitochondrial function.

We confirmed by RT-qPCR that a panel of established genes related to fibrosis and cardiac remodeling, such as fibronectin-1 (*Fn1*), *Cola1*, *Thy1*, *Postn*, and *Pdgfra*, were significantly increased in the hearts of mice exposed to DOX (Figure 7C). The proteins encoded by these genes are suspected to interact with FAP, as evidenced by the connections noted in the STRING database (Supplemental Figure 4G and Supplemental Table 10). In agreement with published reports (40), we observed a 3.8-fold increase in matrix metalloproteinase (MMP-2) expression. This was reinforced by comparable changes in the expression of transcription factor EB (TFEB), which regulates MMP-2 expression and is reported to be a marker of DOX cardiotoxicity (30, 41), and tissue inhibitor of metalloproteinases 2 (TIMP-2), an inhibitor of MMP-2 (Figure 7, D and E). TIMP is also a known substrate of FAP (42). These findings support a possible role for FAP in MMP-2–mediated ECM remodeling in atrophied hearts. Consequently, the increase in cardiac [^^68^^Ga]Ga-FAPI-04 PET signal likely reflects early molecular pathological remodeling induced by DOX.

### *Early FAPI PET predicts subsequent heart growth and fibrosis in male mice in the symptomatic disease phase*.

**Figure 8:**
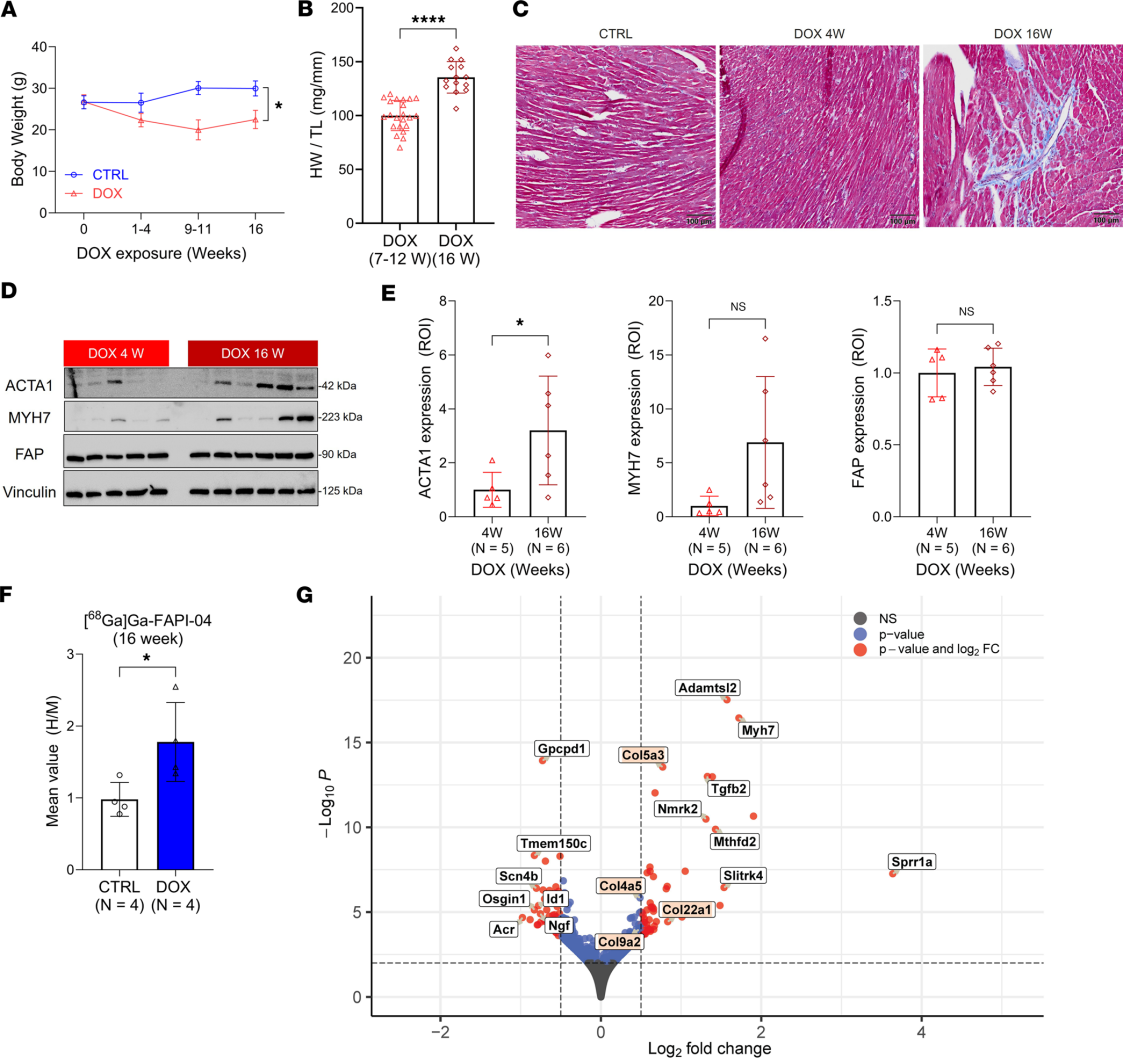
Exposure to DOX induces sustained cardiac tissue remodeling that can result in myocardial fibrosis. (**A**) Comparison of body weight between DOX-treated mice and their controls from 0 to 16 weeks. The number of mice is displayed in Supplemental Table 1. (**B**) HW/TL ratio between DOX groups. DOX (7–12W) group was taken from Figure 1D. (**C**) Representative trichrome staining images of cardiac tissue collected from control mice (10–12 W) and DOX 4W and DOX 16W groups. Corresponding histopathological observations are communicated in Supplemental Table 10. Scale bar: 100 µm. (**D**) Western blot analysis of cardiac ACTA1, MYH7, and FAP expression in tissue taken from mice exposed to DOX after 4 and 16 weeks. Vinculin is used as a reference. (**E**) ROI quantification of each protein level from **D** was performed using ImageJ. (**F**) Image-derived quantitation of cardiac [^68^Ga]Ga-FAPI-04 PET signals normalized to skeletal (calf) muscle uptake (heart/muscle ratio, H/M) at 16 weeks. (**G**) EnhancedVolcano plot from the bulk RNA sequencing at 16 weeks. A total of 49,315 variables were displayed (log2 fold change [FC] > |0.5|, *P* < 0.01). Red boxes: Top upregulated enrichment score genes marked as extracellular matrix organization (GO:0030198; *P* < 0.00045). The top 7 genes with the highest or lowest log2FC values were displayed. These genes and their function are summarized in Supplemental Table 12. Data are presented as the mean ± SD. Statistical comparisons were performed using an unpaired 2-tailed *t* test (**A**, **E**, and **F**), with Welch’s correction in **A**, or a 2-tailed Mann-Whitney test (**B**). *P < 0.05; ****P < 0.0001; ACTA1, skeletal α-actin; MYH7, myosin heavy chain 7; FAP, fibroblast activation protein α; ROI, region of interest.

Following the initial sharp reduction in body weight for the mice in the DOX group, body weights remained steady between 4 and 16 weeks (Figure 8A and Supplemental Table 1). By contrast, the body weights of the control animals continued to increase. In this context, it is notable that the HW/TL ratio difference between the 2 groups decreased over time. At 16 weeks, which we defined to be the late phase of pathology, the HW/TL was approximately 19% lower in the mice exposed to DOX than the controls, which represents a 35% increase compared with the levels observed during the period of acute cardiac atrophy (Figure 1D, Figure 8B, and Supplemental Figure 5A). This finding suggested the possibility of accelerated heart growth in the late disease phase that could indicate the emergence of myocardial fibrosis (43). To this end, we compared interstitial collagen in the left and right ventricle or around the vessel at 4 weeks and 16 weeks and found substantially higher fibrosis in many of the 16-week samples (Figure 8C and Supplemental Table 11). Protein markers of cardiac hypertrophy (44), such as ACTA1 (3.2-fold, *P* = 0.0452) and myosin heavy chain 7 (MYH7) (6.9-fold, *P* = 0.0632), were also expressed at higher levels in cardiac tissue taken from the DOX group at 16 weeks compared with 4 weeks (Figure 8, D and E), though only the change in ACTA1 expression was statistically significant. FAP expression increased but did not significantly change over this interval. Not surprisingly, this was confirmed by [^^68^^Ga]Ga-FAPI-04 PET imaging in the 16-week group (Figure 8F). These findings indicate that early cardiac [^^68^^Ga]Ga-FAPI-04 PET imaging could predict some of the canonical features of DOX-induced cardiotoxicity, including myocardial fibrosis and accelerated cardiac growth.

This conclusion is further supported by bulk RNA-sequencing analysis performed using cardiac tissue collected at 16 weeks. This analysis revealed a substantial reduction in the number of DEGs compared with the analysis at 4 weeks (Figure 8G). Nevertheless, we confirmed that the upregulated genes with the highest enrichment score were associated with ECM organization (Figure 8G, red boxes; Supplemental Figure 5B; and Supplemental Table 12), highlighting the persistent activation of remodeling processes in cardiac tissue exposed to DOX. Moreover, we identified the 7 genes with the highest log2FC values to be associated with processes known to contribute to hypertrophic heart failure (Figure 8G and Supplemental Table 13). Collectively, our findings indicate that male mice exposed to DOX are at risk of developing long-term hypertrophic heart failure and that the molecular changes associated with pathological remodeling, including increased FAP expression, are evident before disease is symptomatic and can be imaged by PET.

## Discussion

In this study, we aimed to validate PET imaging targets that are more sensitive to the early molecular changes associated with DOX-induced cardiotoxicity than conventional echo, while also serving as biomarkers for specific pathophysiological processes. Early diagnosis of cardiotoxicity could greatly improve its treatment, as evidenced by the more complete recovery of LVEF in patients treated with enalapril shortly after anthracycline chemotherapy than in patients treated a few months later (45). Similar outcomes have also been observed with dexrozaxane (46). Nuclear medicine approaches, which primarily image perfusion (47), have been underutilized for this purpose. Recently, new probes have been developed to image specific cardiac pathophysiologies (47). These probes have largely been studied in preclinical models, where they have provided important insight into the development and progression of cardiotoxicity. To build on this work, we selected 3 radioligands, [^^68^^Ga]Ga-FAPI-04 (48), [^^18^^F]DPA-714 (49), and [^^18^^F]MFBG (50), that are already under clinical investigation for other indications to image biochemical processes that are plausibly initiated in response to anthracycline exposure. Preliminary reports indicate that the radiation dosimetry of these probes is acceptable, and therefore they could be used to assess heart health in cancer survivors. We anticipate that this will facilitate the future clinical translation of our imaging approach.

To test our hypothesis, we reproduced a model of DOX-induced cardiotoxicity in male C57BL/6J mice (31, 51). We found, as have others (31), that female mice are less susceptible to DOX exposure than males, leading us to restrict our study to male mice and necessarily limiting the generalizability of our findings. These mice experienced cardiac atrophy upon exposure to DOX and developed left ventricular dysfunction by 10 weeks. These findings are broadly consistent with prior findings in male C57BL/6 mice receiving a similar cumulative dose of DOX (51–54), though there is considerable variability reported between experiments even when the mouse age and strain and dosing regimens are identical. The expression of atrogin1 and MuRF1 in the heart increased shortly after exposure to DOX (Figure 2C). These ubiquitin ligases, which are markers of cardiomyocyte atrophy (28), also support autophagy in these cells (55, 56), which may indicate that metabolic deficiencies in cardiomyocytes drive autophagy as a survival response (30, 57). Upregulation of autophagy would likely result in reduced cardiomyocyte size (58, 59). At 16 weeks, accelerated cardiac growth accompanied by interstitial fibrosis was evident. These features recapitulate important aspects of DOX-induced cardiotoxicity in human patients, for which loss of left ventricular mass is observed at follow-up prior to the emergence of fibrosis and hypertrophic cardiomyopathy (28, 60–63).

One profound change induced by DOX exposure was a sustained increase not only in cardiac remodeling–related makers but also in FAP expression and activity in male mouse hearts, even before cardiac dysfunction was observed. FAP is an attractive target for imaging incipient cardiotoxicity compared with TSPO and NET because of its low basal expression in both mouse and human tissues (Supplemental Figure 6, A and B, and Supplemental Table 14) and its known involvement in tissue remodeling across various pathological conditions (64). Our findings further support this, showing that expression of FAP, but not TSPO and NET, rapidly increased upon exposure to DOX and was sustained from this initial exposure through subsequent cardiac tissue remodeling. Prior work in rat models of myocardial infarction report that FAP expression peaks within 1 week of infarct (65, 66), though persistent FAP activation is evident in C57BL/6 mice for up to 3 weeks after myocardial infarction (67). This highlights possible differences between species that require further investigation. We are not aware of other studies that have longitudinally investigated cardiac FAP expression in mice after exposure to doxorubicin, though a recent study in a rat model confirmed elevated FAP expression 6 weeks — the latest time point studied — after the initial dose (68).

Despite sustained cardiac FAP expression in the mice exposed to DOX, we observed variable levels of fibrosis. It may be that this heterogeneous pattern, which conflicts with some literature reports, is due to the strain of mice, as prior data indicate substantial variability even within strains of C57BL/6 mice (30). Notwithstanding the absence of overt fibrosis before 16 weeks, we found evidence of upregulation of multiple components of cardiac remodeling, such as Fn1 and MMP-2, and genetic signatures characteristic of this process. Given the absence of substantial cardiac fibrosis in our mice in the early disease stage and the possible activation of FAP within cardiomyocytes in addition to myofibroblasts, we hypothesize that hearts exposed to DOX undergo an initial phase of compensatory structural remodeling of the ECM primarily to preserve cell-cell interactions between atrophied cardiomyocytes (69). As disease progresses through the middle phase, the DOX-exposed heart transitions to an accelerated growth profile characterized by expression of genes and proteins (e.g., ACTA1, MYH7) linked to hypertrophy. We did not continue our studies beyond 16 weeks, but it is plausible that the HW/TL ratio of the mice exposed to DOX would continue to increase and eventually exceed the control group. This implies that [^^68^^Ga]Ga-FAPI-04 PET could be used to predict hypertrophy and progression to heart failure in at least male individuals experiencing anthracycline-induced cardiotoxicity and heart disease of different origins. We plan to test this hypothesis in future studies.

To our knowledge, this is the first report that murine and human cardiomyocytes exposed to DOX increase expression of Fap nucleic acid and FAP protein. In cultured HCMs, exposure to DOX alone was sufficient to increase [^^68^^Ga]Ga-FAPI-04 binding, suggesting that this cell population makes a sizable contribution to the elevated cardiac uptake of this tracer in vivo. These findings suggest that FAP may not be exclusive to myofibroblasts, but rather can be activated in cardiomyocytes themselves to contribute to compensatory remodeling in the damaged heart, though this hypothesis requires further testing.

A recent case study speculated that incidental myocardial FAPI PET signal detected in a cancer patient may have been due to cardiotoxicity arising from the chemotherapy regimen (7), although this hypothesis was not explored further. However, the association between high FAP signal and role of FAP in cardiac pathology was described in other cohorts of patients with cardiovascular disease (36). We demonstrated that [^^68^^Ga]Ga-FAPI-04 cardiac SUV increased almost immediately after DOX treatment, substantially earlier than functional deficits were imaged by echo, and was sustained throughout the observation period. PET signal significantly correlated with *Fap* expression in cardiac tissue (Figure 5C) and, additionally, significantly correlated with cardiomyocyte atrophy (Figure 7A).

Due to known sex differences in doxorubicin cardiotoxicity, our findings are not necessarily generalizable. Nevertheless, our work supports the introduction of FAP PET imaging to the clinical care of at least some patients undergoing anthracycline chemotherapy. This potentially addresses a major clinical need, as the development of functional deficits may arise over years and be irreversible at the time of detection. Indeed, interest in cardiac PET imaging is rapidly advancing in cardio-oncology (70) because of its potential to detect critical pathological processes in patients with known risk factors (namely, cancer therapies). The DOX dose administered to these animals translates to a cumulative dose of 72 mg/m^^2^^ in human patients. Currently, the maximum recommended dose is 450 mg/m^^2^^ for adults (71), while pediatric patients may experience cardiotoxicity at cumulative doses less than 300 mg/m^^2^^ (72). Even at this low dose level, we detected increased cardiac SUV immediately upon cessation of DOX treatment. This finding highlights the sensitivity of [^^68^^Ga]Ga-FAPI-04 PET approach to incipient, presymptomatic disease. We present a biochemical rationale for the targeting of FAP for diagnostic or prognostic imaging approaches. In contrast with other markers of tissue remodeling, which increase at earlier time points and return to baseline by 16 weeks, upregulation of FAP is sustained throughout disease progression, resulting in a broad diagnostic window. This may reflect the contribution of different cell types to increased FAP signal, with early upregulation in cardiomyocytes transitioning to later expression in the activated fibroblasts that contribute to myocardial fibrosis. Moreover, serum FAP levels were higher in control mice compared with DOX-treated animals, indicating that circulating FAP is not a reliable biomarker in this model. Recent studies in clinical populations with cardiovascular disease similarly found no correlation between circulating FAP levels and tissue FAP expression (33, 34, 73). As tissue remodeling is pathological in some forms of cardiac disease, FAP has been proposed to be a potential drug target (33). Consequently, methods of detecting its expression noninvasively may have broader application beyond cardiotoxicity. These factors lead us to propose that detection of FAP expression by PET imaging could serve as a key diagnostic and prognostic biomarker of early disease and a method of evaluating response to treatment in heart failure.

In contrast with FAP, our alternative molecular targets, TSPO and NET, did not prove to be biomarkers of DOX-induced cardiotoxicity. Our rationale for targeting TSPO was the prominent role that oxidative stress and inflammation play in DOX-induced cardiotoxicity (17), but we observed neither a significant increase in [^^18^^F]DPA-714 signal nor an increase in TSPO staining by immunohistochemistry. Moreover, we found no evidence of macrophage infiltration by histology or CD11b immunohistochemistry (Supplemental Figure 3C). A previous study reported decreased uptake of radiolabeled *meta*-iodobenzylguanidine in the hearts of rats treated with anthracyclines (74), but we found no change in cardiac [^^18^^F]MFBG signal in the hearts of the DOX-treated mice. These observations highlight the sensitivity of FAP PET as an indicator of incipient cardiotoxicity caused by DOX.

We acknowledge several limitations of our study. First, our imaging studies show elevated background [^^68^^Ga]Ga-FAPI-04 PET signal in DOX mice, even though there were no significant differences in SUVmax of skeletal muscle between the different imaging time points. This likely reflects tissue remodeling occurring in other organs, such as lung and skeletal muscle, induced by systemic administration of DOX. Global signal increase was previously observed in FAPI PET imaging of a preclinical model of idiopathic pulmonary fibrosis induced by bleomycin (75). Second, although we have demonstrated that the cardiac [^^68^^Ga]Ga-FAPI-04 PET signal is associated with cardiac atrophy and likely predicts functional decline and later interstitial fibrosis, in light of the upregulation of FAP by cardiomyocytes, the specific role that FAP plays in the remodeling process induced by DOX remains unknown. As such, a definitive causal relationship remains to be established between FAP expression and subsequent cardiac dysfunction, and we cannot rule out the possibility that DOX-induced increases in FAP are unrelated to cardiac dysfunction. Causality could be investigated by experimental manipulation of FAP expression, with PET imaging and echo used to determine the effect of these manipulations on cardiac function. Finally, we were unable to investigate sex as a biological variable due to the resistance of female C57BL/6J mice to DOX treatment. As such, our results may not be universally applicable across the spectrum of patients with cancer.

In summary, we report a significant and sustained increase in FAP expression in male mice immediately following systemic administration of doxorubicin. This change is detectable using PET imaging with [^^68^^Ga]Ga-FAPI-04 even before the onset of cardiac dysfunction. Notably, we provide biochemical rationale for targeting FAP as an imaging biomarker and report for the first time to our knowledge its upregulation in cardiomyocytes exposed to DOX. The sustained upregulation of FAP expression throughout the symptomatic phase in which we observed accelerated growth highlights its suitability for predicting hypertrophy and myocardial fibrosis. These findings indicate that FAPI PET could serve as a valuable complement to echo in managing patients with cancer undergoing anthracycline chemotherapy. Moreover, its association with cardiac tissue remodeling in various forms of heart failure highlights its potential as an imaging biomarker for these conditions as well.

## Methods

### *Sex as a biological variable*.

Our study examined male mice because female mice of this species and strain are less susceptible to doxorubicin-induced toxicity. It is not known whether our findings apply to female mice.

### *General*.

A full description of the methods, including synthesis of [^^68^^Ga]Ga-FAPI-04, [^^18^^F]DPA-714, and [^^18^^F]MFBG; echocardiography and microPET/CT imaging and analyses; tissue preparation; Western blot analyses; quantitative RNA analyses; histological and immunohistochemical analyses; FAP activity determination; and bioinformatic analyses, can be found in the Supplemental Methods.

### *Animal studies*.

Adult male C57BL/6J (8-week-old) mice were purchased from The Jackson Laboratory and randomly assigned to treatment (*n* = 64) or control (*n* = 25) groups. Mice in the treatment group were administered a solution of doxorubicin in saline at 3 mg/kg every other day for 2 weeks (total 8 doses; cumulative dose of 24 mg/kg) by intraperitoneal (i.p.) administration (51). Age-matched control mice were administered the same volume of saline i.p. Mice were weighed every other day for 4 weeks after the first administration and subsequently once weekly. Criteria for euthanasia were decrease in body weight > 20% and prolonged hunched posture with or without difficulty feeding. The mice were randomly assigned to be imaged by echocardiography (weeks 4 and 10) or PET on a weekly basis for a total of 16 weeks. Mice were sacrificed at the PET imaging time points, and hearts were perfused and isolated to provide tissue for ex vivo analysis.

### *Statistics*.

Statistical analyses were carried out using GraphPad Prism 10.1.2. All data were expressed as means ± SD and are representative of at least 3 separate biological experiments. The unpaired 2-tailed Student’s *t* test or Mann-Whitney *U* test was used for comparisons of 2 groups. For comparison between 3 or more groups, 1-way ANOVA with Tukey’s multiple comparisons was performed. For DEG analysis, corrected *P* values were calculated based on the Benjamini-Hochberg method to adjust for multiple testing. For correlation analysis, the Pearson correlation test was used. A *P* value of less than 0.05 was considered statistically significant, with the exception of the DEG analysis, for which *P* < 0.01 was used as the signifier of statistical significance.

### *Study approval*.

All animal studies were approved by the Institutional Animal Care and Use Committee of Weill Cornell Medicine (protocol number 2019-0043) and were undertaken in accordance with the guidelines set forth by the 2015 US Public Health Service *Policy on Humane Care and Use of Laboratory Animals*.

### *Data availability*.

Underlying data summarized in the manuscript figures can be found in the Supporting Data Values XLS file. Additional data, such as image DICOM files, are available from the authors upon request.

## Author contributions

The studies were conceived by JMK, JB, and ADL. Experiments were designed by CHL and JMK and performed by CHL, OLM, LR, and TMJ. Data analysis was performed by CHL, OLM, SC, TMJ, ADL, and JMK. Pathology and quantitation of ISH and IHC were performed by SEC. Funding was acquired by JMK. The manuscript was written by CHL and JMK and reviewed by OLM, LR, SEC, SC, TMJ, JB, and ADL.

## Funding support

This work is the result of NIH funding, in whole or in part, and is subject to the NIH Public Access Policy. Through acceptance of this federal funding, the NIH has been given a right to make the work publicly available in PubMed Central.

National Cancer Institute (NCI) at the NIH (R21CA246409 to JMK).

NCI Cancer Center Support Grant P30CA008748 issued to Memorial-Sloan Kettering Cancer Center (Laboratory of Comparative Pathology, SEC).

## Supplementary Material

Supplemental data

Unedited blot and gel images

Supporting data values

## Version 1 10/23/2025

In-Press Preview

## Version 2 12/08/2025

Electronic publication
