# Supplementary material for: FAP PET identifies earlycardiac molecular changesinduced by doxorubicin chemotherapy
Source: JCI Insight. 2025 Oct 23;10(23):e191058. doi: 10.1172/jci.insight.191058 (PMC12890471; doi:10.1172/jci.insight.191058)
Supplement: Supplemental data [file jciinsight-10-191058-s037.pdf]

SUPPLEMENTAL INFORMATION

FAP PET Identifies Early Cardiac Molecular Changes Induced by Doxorubicin Chemotherapy

Chul-Hee Lee[1], Onorina L. Manzo[2,3,4], Luisa Rubinelli[2,3,4], Sebastian E. Carrasco[5,6,7,8], Sungyun Cho[9,10,13], Thomas M. Jeitner[1], John W. Babich[1,10,11,12], Annarita Di Lorenzo[2,3,4], James M. Kelly[1,10,11*]

[1]Molecular Imaging Innovations Institute and Department of Radiology, Weill Cornell Medicine, New York, NY

[2]Department of Pathology and Laboratory Medicine, Weill Cornell Medicine, New York, NY

[3]Cardiovascular Research Institute, Weill Cornell Medicine, New York, NY

[4]Brain and Mind Research Institute, Weill Cornell Medicine, New York, NY

[5]Laboratory of Comparative Pathology, Weill Cornell Medicine, New York, NY

[6]Laboratory of Comparative Pathology, Memorial-Sloan Kettering Cancer Center, New York, NY

[7]Laboratory of Comparative Pathology, Rockefeller University, New York, NY

[8]Laboratory of Comparative Pathology, Hospital for Special Surgery, New York, NY

[9]Department of Pharmacology, Weill Cornell Medicine, New York, NY

[10]Sandra and Edward Meyer Cancer Center, Weill Cornell Medicine, New York, NY

[11]Citigroup Biomedical Imaging Center, Weill Cornell Medicine, New York, NY

[12]Present address: Ratio Therapeutics, Inc. Boston, MA

[13]Present address: Department of Pharmacology, Korea University College of Medicine, Seoul, South Korea

CONTENTS

Supplemental Methods……………………………………………………...…………..…2

References………………………………………………………………………….…..….. 10

Supplementary Figures…………………………………………………………………….…..11

Supplementary Tables……………………………………………………………...……….22

Supplemental Methods

General

Doxorubicin hydrochloride was purchased from Tocris Bioscience, USA and used without further purification. It was dissolved at a concentration of 0.75 mg/mL in sterile saline for injection (Hospira, USA) with the aid of sonication. The solution was stored in the dark at -20 °C for up to 24 h before use.

Echocardiography imaging and analysis

Cardiac dimensions and function were determined by transthoracic echocardiography according to previously published methods (1). Mice were imaged individually under inhaled isoflurane anesthesia on a 37 °C heated platform. Scans were acquired using left-ventricle M-mode and all measurements were obtained by averaging the values of three consecutive cardiac cycles. Left-ventricle end-diastolic (LVDd) and end-systolic (LVDs) dimensions were measured using M-mode traces. Fractional shortening (FS) was calculated using the formula [(LVDd-LVDs)/LVDd]. Diastolic measurements were estimated at the point of maximum cavity dimension, and systolic were taken at the point of minimum cavity dimension, according to the American Society of Echocardiography's recommended method (2). The average heart rate was determined by calculating the distance over time between cardiac cycles of three consecutive cardiac cycles.

Radiosynthesis

General

(*S*)-2,2',2''-[10-[2-[4-[3-[[4-[[2-(2-Cyano-4,4-difluoro-1-pyrrolidinyl)-2-oxoethyl]carbamoyl]-6-quinolyl]oxy]propyl]-1-piperazinyl]-2-oxoethyl]-1,4,7,10-tetraazacyclododecane-1,4,7-triyl]triacetic Acid (FAPI-04) was purchased from Millipore Sigma, USA and used without further purification. It was dissolved to a concentration of 1 mg/mL in dimethyl sulfoxide (DMSO; Millipore Sigma). Toluene-4-sulfonic acid 2-[4-(3-diethylcarbamoylmethyl-5,7-dimethyl-pyrazolo-[1,5-a]pyrimidin-2-yl)-phenoxy]-ethyl ester, the precursor for [^{18}F]DPA-714, and *N,N*-diethyl-2-(2-[4-(2-fluoroethoxy)-phenyl]-5,7-dimethyl-pyrazolo[1,5-a]pyrimidin-3-yl)-acetamide (DPA-714) were synthesized by the Millstein Core Chemistry Facility at Weill Cornell Medicine according to published procedures (3) (4-Methoxyphenyl)(3-((1,2,3,3-tetrakis(*tert*-butoxycarbonyl)guanidino)methyl)phenyl)-iodonium trifluoromethanesulfonate (ALP-mFBG) was synthesized according to published procedures (4) and gifted by Stephen G. DiMagno (University of Illinois-Chicago, Chicago, USA). MFBG hydrochloride, the non-radioactive standard, was gifted by Illumina Radiopharmaceuticals (New York, NY, USA). ALP-mFBG was dissolved to a concentration of 7.5 mg/mL in anhydrous acetonitrile (MeCN; Millipore Sigma). All other chemicals and reagents were purchased from Millipore Sigma and used without purification unless otherwise indicated.

Analytical and semi-preparative high-performance liquid chromatography (HPLC) were performed on a dual pump Varian high-performance liquid chromatography system (Agilent Technologies, USA) fitted with a dual ultraviolet-visible light (UV–vis) detector and an NaI(Tl) detector (Bioscan, USA).

[^{68}Ga]Ga-FAPI-04

The synthesis of [^{68}Ga]Ga-FAPI-04 was adapted from previously reported methods (5) [^{68}Ga]$GaCl_3$ was eluted from a ^{68}Ge/^{68}Ga generator (ITM, Germany) in 4 mL of a 0.05 M hydrochloric acid solution. Two mL of the eluate was added to a plastic centrifuge tube (VWR, USA) containing 20 µL of a 1 mg/mL solution of FAPI-04 in DMSO. The pH was adjusted to approximately 4 by addition of 40 µL 3 N sodium acetate and the reaction was heated to 95 °C for 15 min on a ThermoMixer® C (Eppendorf, USA). The reaction was reformulated using a Sep-Pak C18 light cartridge (Waters, USA) and typically obtained as a 200 MBq/mL solution in 10% *v/v* ethanol/saline. Radiochemical purity (RCP) was determined by analytical HPLC using a dual-pump Varian Dynamax HPLC system (Agilent Technologies, USA) fitted with a dual UV-Vis detector and a NaI (Tl) flow count detector (BioScan, Inc., USA). UV absorption was monitored at 220 nm and 280 nm. Analytes were injected on to a Symmetry C18 (5 µm, 4.6 × 50 mm, 100 Å) column (Waters, USA) and a gradient mobile phase run at the following schedule: 0% B from 0-2 min, 0-100% B from 2-8 min, 100% B from 8-9 min, and 100-0% B from 9-10 min. Solvent A was water + 0.01 % trifluoroacetic acid (TFA) and solvent B was 90% *v/v* acetonitrile/water + 0.01% TFA. RCP exceeded 99%. The flow rate was 2 mL/min. The molar activity of the reformulated product was typically 15-20 GBq/µmol.

[^{18}F]DPA-714

The synthesis of [^{18}F]DPA-714 was adapted from previously reported methods (3). Fluorine-18 was obtained by irradiation of $H_2{}^{18}O$ (Rotem Industries, Israel) via the ^{18}O(p,n)^{18}F transformation using a TR19 cyclotron (Advanced Cyclotron Systems, Inc., Canada). End-of-bombardment activity was typically 3.7-7.4 GBq. [^{18}F]Fluoride was trapped on a QMA light carbonate cartridge (Waters, USA) and eluted with 1 mL of an 80% *v/v* MeCN/water solution containing 10 mg kryptofix-222 and 5 mg potassium carbonate. The mixture was dried azeotropically and 0.5 mL of a 6 mg/mL solution of tosylate precursor in MeCN was added to the reaction vial. The reaction was heated at 85 °C for 10 min. The crude product was diluted with 1 mL water and purified by semi-prep HPLC following injection on a PRP-1 (7 µm, 10 x 250 mm, 100 Å) column (Hamilton, USA) and using a gradient mobile phase from 0-100% B over 20 min. Solvents A and B were as above. The flow rate was 8 mL/min. [^{18}F]DPA-714 was reformulated using a Sep-Pak C18 light cartridge and typically eluted as a 300 MBq/mL solution in 10% *v/v* ethanol/water. RCP was verified by analytical HPLC as above and exceeded 99%. The molar activity of the reformulated product was approximately 110 GBq/µmol.

[^{18}F]MFBG

The synthesis of [^{18}F]MFBG was adapted from previously reported methods (6). [^{18}F]Fluoride was obtained and trapped on a QMA light carbonate cartridge as described above. End-of-bombardment activity was typically 9.25-12 GBq. The fluoride was eluted with 0.5 mL of a 50% *v/v* MeCN/water mixture containing 10 mg cesium carbonate. The mixture was dried azeotropically before 800 µL of a 7.5 mg/mL solution of ALP-mFBG in MeCN was added. The reaction was heated at 140 °C for 12 min in a microwave reactor (Biotage, USA). The solvent was then evaporated at 100 °C and the crude residue was suspended in 1 mL 2 M hydrochloric acid and heated for 10 min at 140 °C in the microwave reactor. The crude mixture was diluted with 1 mL water and purified by semi-prep HPLC following injection onto a PRP-1 (7 µm, 10 x 250 mm, 100 Å) column (Hamilton, USA). The column was eluted using an isocratic mobile phase consisting of 10% v/v ethanol/25 mM hydrochloric acid at a flow of 6 mL/min. The pH of the

collected fraction was adjusted to 5-6 with 1 M ammonium acetate, pH 7. [^{18}F]MFBG was typically obtained in concentration of 50 MBq/mL. RCP was verified by analytical HPLC as above and exceeded 98%. Molar activity was approximately 100 GBq/µmol.

Small animal microPET/CT imaging

Mice were intravenously administered 3.7-11.1 MBq of the corresponding radioligand in a total volume of 100-150 µL. Imaging was performed using small animal microPET/CT (Siemens Inveon™, USA) under isoflurane anesthesia (3.5% for induction, 1.5 % for maintenance) beginning 45 min after injection. The total PET acquisition time was 30 min, and a CT scan was obtained immediately before the PET acquisition for anatomic co-registration and attenuation correction. Data was collected in list mode and images were reconstructed using the OSEM-MAP algorithm.

MicroPET/CT image analysis

All microPET/CT images were evaluated with the AMIDE algorithm (A Medical Image Data Examiner) (7). An ellipsoidal volume of interest (VOI) was generated for the heart and the right calf muscle (25 ± 2 mm^3) using the CT image. Representative coronal maximum intensity projections (MIPs) with the VOI indicated are depicted in Supplemental Figures 2K and L. The mean counts in the VOI were converted to the percent injected dose per cubic centimeter (%ID/cm^3) and calibrated against a 1% injected dose standard. The %ID/cm^3 in the heart was normalized to the %ID/cm^3 in the muscle, providing a heart/muscle ratio, H/M. Mean and maximum standardized uptake values (SUVmean and SUVmax) were determined by dividing the ratio of heart activity concentration to total injected dose by the body mass of the mouse: SUV = $A_{ROI}/A_{inj}/BW$.

Tissue-based quantification of cardiac [^{68}Ga]Ga-FAPI-04 uptake

DOX mice at 4 weeks and age-matched control groups (n = 4 per group) were injected intravenously with a bolus injection of 1.1-2.5 MBq [^{68}Ga]Ga-FAPI-04 containing 0.2 µg of FAPI-04. The mice were sacrificed at 1 h post-injection. The hearts were excised, exsanguinated, weighed, and counted on a 2470 Wizard^2 automatic γ-counter (Perkin Elmer. USA). Counts were corrected for decay and activity injected, and tissue uptake was determined by comparison with a known standard representing 1 % of the injected dose (ID). Cardiac activity was normalized by heart mass to provide activity concentrations expressed as %ID/g ± standard error of the mean (SEM).

Mouse serum FAP ELISA assay

Mouse blood (CTRL and DOX groups, n = 4 each) was collected at 4 weeks by cardiac puncture. The assay was carried out according to the protocol provided by the manufacturer (#ab289903, ThermoFisher, USA). Briefly, blood samples were centrifuged at 2,000 x g for 10 min, and the fraction containing the serum was collected. Samples were detected at 450 nm. The concentration of serum FAP (pg/mL) was calculated by the same protocol provided by the manufacturer.

Preparation of heart tissue for ex-vivo analysis

The mice were anesthetized by i.p. ketamine injection and perfused with phosphate-buffered saline (PBS) via the left ventricle at a constant pressure of 80 mmHg. The hearts were patted dry and weighed on a digital balance. To perform the molecular and histological analysis, the hearts were

cut transversally at the mid-horizontal plane. Cut fractions were separated for RNA and protein extraction, and tissue staining. Regions for tissue staining were fixed overnight in formalin at 4 °C and stored in 70% ethanol until further processing. The fractions for RNA and protein extraction were flash frozen in liquid nitrogen and stored at -78 °C until further use.

Heart weight to tibia length (HW/TL) ratios

The tibia was collected ex vivo and all soft tissue was removed. The length was measured using a digital caliper. Heart weights were determined as described above and divided by the corresponding tibia length from the same animal to produce the heart weight to tibia length (HW/TL) ratio.

Western blot analysis

Frozen heart tissue was pulverized by mortar and pestle for tissue homogenization. Ground heart tissue powders were collected and soaked in tissue protein extraction reagent (#78510, ThermoFisher, USA) supplemented with a protease inhibitor cocktail (#87786, ThermoFisher, USA) for protein extraction. Protein concentrations and western blot (WB) were performed as previously reported (8). The information on primary and secondary antibodies was listed in the supplemental table of antibodies. The chemical luminescent signals were measured by Azure c400 Gel imaging system (Azure Biosystems, Inc. USA) and ChemiDoc imaging system (Bio-Rad, USA). Protein expression was quantified by drawing a region of interest (ROI) using ImageJ software.

Quantitative RT-PCR analysis

Ground heart tissue powders were collected and soaked in Trizol (Invitrogen, USA) and RNeasy Fibrous tissue mini kit (Qiagen, USA) was used to isolate total RNA from heart tissues. Genomic DNA was removed by DNase I (Qiagen), and RNA was reverse transcribed using an iScript kit (Bio-Rad, USA). The resulting cDNA was analyzed by quantitative RT-PCR (qPCR) using SYBR green master mix (Life Technologies, USA) on QuantStudio6 Real-Time PCR system (Life Technologies). mRNA levels were calculated by the delta-delta CT method of the target gene (Atrogin1, *Murf1*, *Fap*, and *Tspo*) based on independent primer sets and four reference genes (*Rpl32*, *Tbp*, *Gapdh*, and *Actb*). The full primer list is reported in Supplemental Table 5.

Bulk RNA-seq library construction and data analysis

RNA libraries were sequenced with paired-end 50 bps on the NovaSeq 6000 Sequencer (Illumina, USA). The raw sequencing reads in BCL format was processed through bcl2fastq 2.20 (Illumina) for FASTQ conversion and demultiplexing. After trimming the adaptors with cutadapt (version 1.18; https://cutadapt.readthedocs.io/en/v1.18/), RNA reads were aligned and mapped to the GRCm39 mouse reference genome by STAR (version 2.5.2; https://github.com/alexdobin/STAR) (9), and transcriptome reconstruction was performed by Cufflinks (Version 2.1.1) (http://cole-trapnell-lab.github.io/cufflinks/). The abundance of transcripts was measured with Cufflinks using fragments per kilobase of transcript per million mapped reads (FPKM) as an output (10, 11). Raw read counts per gene were extracted using HTSeq-count version 0.11.2 (12). Gene expression profiles were constructed for differential expression, cluster, and principle component analyses with the DESeq2 package (https://bioconductor.org/packages/release/bioc/html/DESeq2.html) (13). For differential expression analysis, pairwise comparisons were performed between two or more groups using parametric tests where read counts follow a negative binomial distribution with

a gene-specific dispersion parameter. Corrected *p*-values were calculated based on the Benjamini-Hochberg method to adjust for multiple testing. For the differentially expressed genes (DEGs) analysis at 4 weeks group, $p < 0.01$ was used as the signifier of statistical significance, and Log2FC (FC, fold change) ≥ 0.55 and Log2FC ≤ -0.85 were used to distinguish upregulated (Up) and downregulated (Down) DEGs, respectively. In the case of the 16 weeks, we divided DEGs within false discovery rate (FDR)<0.05 into Up and Down groups for analysis. EnhancedVolcano plot and heatmap clustering was generated using R studio to compare DEGs between groups and for the overall distribution of DEGs

DAVID database

The Database for Annotation, Visualization, and Integrated Discovery (DAVID) was used to group DEGs based on biological function (https://david.ncifcrf.gov/). The 1326 upregulated and 1684 downregulated genes in the 4-week group, and the 86 upregulated and 107 downregulated genes in the 16-week group were submitted for the Gene Ontology (GO) according to the biological process (BP) and KEGG pathway analyses. GO terms with a *p*-value cutoff < 0.05 were regarded as significant.

STRING database

A protein-protein interaction (PPI) network was developed to identify the association between a target and related DEGs by utilizing the search tool for the retrieval of interacting genes/proteins (STRING) database (http://string-db.org/) (14). GO terms and PPI networks with a p-value cutoff < 0.05 were regarded as significant.

Histopathology

Heart tissue was processed in alcohol and xylene and embedded in paraffin. Four transverse sections of the heart per mouse, including right and left ventricles, right and left auricles, and interventricular septum were sectioned at 5-µm thickness and stained with hematoxylin and eosin. Histopathological evaluation of the heart was performed by a board-certified veterinary pathologist. Hearts were evaluated microscopically and scored on a scale of 1 (minimal) to 4 (marked) on the basis of cardiomyocytes showing necrosis, degeneration, atrophy, leukocytic cell infiltrates, and interstitial fibrosis in agreement with consensus practices (15). Cytoplasmic vacuolization was not used as a criterion for diagnosing cardiomyocyte degeneration as this feature as also observed in control heart tissue.

Formalin-fixed sections of the heart were stained with Masson's Trichrome to evaluate the presence of collagen in cardiac tissues. To determine the percentage of collagen in the heart, digital whole slide images of Masson's trichrome-stained hearts were manually annotated and then classified pixels were evaluated with a random forest algorithm using QuPath (an open-source software for digital pathology image analysis accessed through: https://qupath.github.io/). Regions of collagen for this analysis included collagen fibrils between cardiomyocytes and around preexisting vasculature within cardiac musculature. Regions excluded for this analysis included preexisting collagen from great vessels, leaflet insertion bands, and pericardial connective tissue.

Morphometric analysis of cardiomyocyte size

Cardiomyocyte size was evaluated from the H&E-stained histological images and analysis was performed using the CmyoSize workflow method (16). Briefly, the analogous regions of the

interventricular septum (3 fields) and left ventricle (3 fields) were designated and reviewed by a pathologist (S.E.C.). The regions of the heart in which the myofibers were oriented transversely were identified and size of thirty randomly selected cells from each region was measured. ImageJ software was used for the measurement of cardiomyocyte size (μm^2).

In situ hybridization (ISH)

Formalin-fixed, paraffin-embedded cardiac sections were incubated with the target probe designed to detect region 486 - 1588 of murine fibroblast activation protein (Fap) mRNA, NCBI Reference Sequence NM_007986.3 (RNAscope® LS 2.5 probe for murine *Fap*, #423888; Advanced Cell Diagnostics, Newark, CA). The target probe was validated on sections of murine skin and heart from mice. Slides were stained on an automated stainer (Leica Bond RX, Leica Biosystems) with RNAscope 2.5 LS Assay Reagent Kit-Red (322150, Advanced Cell Diagnostics) and Bond Polymer Refine Red Detection (DS9390, Leica Biosystems). Control probes detecting a validated positive housekeeping gene (mouse peptidylprolyl isomerase B, *Ppib,* to confirm adequate RNA preservation and detection; 313918, Advanced Cell Diagnostics) and negative control, *Bacillus subtilis* dihydrodipicolinate reductase gene, *dapB,* to confirm absence of nonspecific labeling; 312038, Advanced Cell Diagnostics) were used. Positive RNA hybridization was identified as discrete, punctate chromogenic red dots under bright field microscopy. Images were acquired with an Olympus VS200 slide scanner with a 40x objective. Quantitative image analysis of *Fap* hybridization was performed with QuPath using an algorithm for singleplex chromogenic RNAscope image analysis. Fap positive hybridization signal was classified as follows: 1 red dot / cell, 2 red dot / cell, and 3+ red dots / cell in each transverse section of the heart. An H-score of *Fap* positive signal from each sample was calculated by the QuPath software. Samples with autolysis or regions in the tissue with pale-brown precipitate and/or folding artifacts were excluded from this analysis.

Immunohistochemistry (IHC)

Formalin-fixed, paraffin-embedded sections were stained using an automated staining platform (Leica Bond RX, Leica Biosystems). Following deparaffinization and heat-induced epitope retrieval in a citrate buffer at pH 6.0, the primary antibody against TSPO, also known as peripheral-type benzodiazepine receptor (PBR; ab109497, Abcam, Waltham, MA), was applied at a dilution of 1:10000. A rabbit anti-goat secondary antibody (Cat. No. BA-5000, Vector Laboratories, Burlingame, CA) and a polymer detection system (DS9800, Novocastra Bond Polymer Refine Detection, Leica Biosystems) was then applied to the tissues. The chromogen used was 3,3'-diaminobenzidine tetrachloride (DAB) and the sections were counterstained with hematoxylin and examined by light microscopy. Positive immunoreactivity for TSPO was confirmed with internal mouse tissue array controls used to validate this immunoassay. A subset of tissues incubated with antibody diluents and secondary antibody only were used as negative controls for this assay. Images were acquired with an Olympus VS200 slide scanner (Olympus, Tokyo, Japan) with a 20x objective. Quantitative image analysis was performed by using the QuPath Pixel classifier module. A random forest algorithm was used for identifying pixels as TSPO-positive, TSPO-negative and background in cardiac sections. Region of interest and thresholding values were validated by a board-certified veterinary pathologist.

For CD11b immunohistochemistry, a heat-mediated antigen retrieval with citrate buffer (pH 6.0) was applied on deparaffinized cardiac sections, which were then incubated with a primary anti-CD11b antibody at a dilution of 1:4000 (ab133357, Abcam, USA). A goat anti-rabbit secondary

antibody (Cat. No. BA-1000, Vector Laboratories) and a polymer detection system (DS9800, Novocastra Bond Polymer Refine Detection, Leica Biosystems) were then applied to the tissues. The chromogen was DAB, and the sections were counterstained with hematoxylin and examined by light microscopy.

Vimentin and alpha-smooth muscle actin (α-SMA) IHC assays were optimized and validated by the Laboratory of Comparative Pathology. Following deparaffinization and heat-induced epitope retrieval in citrate buffers at pH 6.0 (α-SMA IHC) or at pH 9.0 (vimentin IHC), the primary antibody against vimentin (5741, Cell Signaling, Danvers, Massachusetts) or α-SMA (ab32575, Abcam, USA) was applied at dilutions of 1:250. A goat antirabbit secondary antibody (Cat. No. BA-4001, Vector Laboratories, Burlingame, California) and a polymer detection system (DS9800, Novocastra Bond Polymer Refine Detection, Leica Biosystems) were then applied to the tissues. The chromogen was 3,3'-diaminobenzidine tetrachloride (DAB), and the sections were counterstained with hematoxylin and examined by light microscopy. Positive immunoreactivity for vimentin and α-SMA was confirmed with internal mouse tissue array controls used to validate these immunoassays. A subset of tissues incubated with antibody diluents and secondary antibody only were used as negative controls for these assays.

Tissue FAP activity assay

All reagents were purchased from Sigma Aldrich, USA unless otherwise noted. Tissue extracts obtained at 4 weeks (n = 2) and 5 weeks (n = 1) were prepared in a buffer consisting of 50 mM Tris, 1 M NaCl, 0.5 mM EDTA and Thermo Scientific™ Halt™ Protease Inhibitor Cocktail (ThermoFisher, USA). All extraction steps were performed on ice. The cardiac tissues were first minced using a scalpel blade and then disrupted using a mechanical disruptor (Benchmark D1000 Homogenizer, USA) for 15 s, taking care not to allow the samples to heat. The disrupted tissues were then sonicated for 10 cycles of 30 s sonication and 30 s of cooling using a Biodisruptor (Diagenode, USA). Finally, the extracts were centrifuged for 5 min at 600 x g at 4 °C and the supernatant was collected and used for the assays. The protein content of the supernatants was determined using the BCA assay (ThermoFisher, USA).

FAP activity was measured by quantifying the amounts of 7-methoxycoumarin-4-acetic acid (MCA) released from MCA-Ala-Ser-Gly-Pro-Ala-Gly-Ala-EDDnp (Bachem, Switzerland) as catalyzed by FAP. MCA produces an optimum fluorescence at 393 nm following excitation at 322 nm which was used to measure its release from MCA-Ala-Ser-Gly-Pro-Ala-Gly-Ala-EDDnp. The reaction buffer consisted of 50 mM Tris, 1 M NaCl, 1 mg/mL bovine serum albumin (BSA), 5 µM MCA-Ala-Ser-Gly-Pro-Ala-Gly-Ala-EDDnp, and 50 µg tissue extracts (described above) contained in a volume of 150 µL. Reactions began with the combination of equal volumes of substrate and enzyme mixtures at room temperature, which was typically 22°C. Each set of reactions also included triplicate 150 µL volumes consisting of 50 mM Tris, 1 M NaCl, 1 mg/mL BSA, and 4 mM MCA that were used to calculate the amounts of MCA released during the reactions. The reactions were performed using black walled 96 multiwell plates and a Tecan Infinite M1000 PRO spectrometer (Tecan, Switzerland). Raw fluorescence data were converted to nmol/min/mg.

Effect of DOX on human cardiomyocytes

General

Human cardiomyocytes isolated from adult left ventricles (PromoCell, Heidelberg, Germany) were cultured according to the manufacturer's instructions. In brief, once cultures reached 80-90% confluency, the cells were washed with PBS and fresh medium, with ("two hit") or without (controls, "one hit") 0.1 μM DOX, was added. The cells were incubated for 48 h and then fresh medium was added. After 48 h incubation, the medium was changed and fresh medium, with ("one hit," "two hit") or without (controls) 0.1 μM DOX was added. The cells were incubated for 48 h, at which point the medium was removed and the free medium was added (17). Cell uptake or western blot was performed on day 7.

[^{68}Ga]Ga-FAPI-04 uptake

Human cardiomyocytes were prepared in 24-well plates. On day 7, the cells were washed with PBS and incubated with labeling medium (containing 1.16 MBq of [^{68}Ga]Ga-FAPI-04, molar activity 6.8 GBq/µmol, in cultured medium) in each well for 1 h at 37 °C. Then, the cells were washed twice with PBS and lysed with 1% sodium dodecyl sulfate (SDS). Radioactivity of the cell lysates was counted on a 2470 Wizard^2 automatic γ-counter (Perkin Elmer. USA). Counts were corrected for decay and activity treated, and cell uptake was normalized with the amount of total protein at the time of the assay (% of uptake/mg, %/mg). Total protein concentrations were determined with BCA protein assay kit (ThermoFisher, USA).

Western blot

The human cardiomyocytes were harvested on day 7 to evaluate the FAP and alpha-actinin-1 (ACTN1) expression. Cells were lysed in radio-immunoprecipitation assay (RIPA) buffer (Millipore Sigma) containing a protease inhibitor cocktail for protein extraction. All further steps were identical to the tissue western blot process. The information on primary and secondary antibodies was listed in the supplemental table of antibodies.

References

1. Sasset L, et al. Nogo-A reduces ceramide de novo biosynthesis to protect from heart failure. *Cardiovasc Res.* 2023;119(2):506-19.
2. Mitchell C, et al. Guidelines for Performing a Comprehensive Transthoracic Echocardiographic Examination in Adults: Recommendations from the American Society of Echocardiography. *J Am Soc Echocardiogr.* 2019;32(1):1-64.
3. James ML, et al. DPA-714, a new translocator protein-specific ligand: synthesis, radiofluorination, and pharmacologic characterization. *J Nucl Med.* 2008;49(5):814-22.
4. Hu B, et al. A practical, automated synthesis of meta-[(18)F]fluorobenzylguanidine for clinical use. *ACS Chem Neurosci.* 2015;6(11):1870-9.
5. Lindner T, et al. Development of Quinoline-Based Theranostic Ligands for the Targeting of Fibroblast Activation Protein. *J Nucl Med.* 2018;59(9):1415-22.
6. Pandit-Taskar N, et al. Biodistribution and Dosimetry of (18)F-Meta-Fluorobenzylguanidine: A First-in-Human PET/CT Imaging Study of Patients with Neuroendocrine Malignancies. *J Nucl Med.* 2018;59(1):147-53.
7. Loening AM, and Gambhir SS. AMIDE: a free software tool for multimodality medical image analysis. *Mol Imaging.* 2003;2(3):131-7.
8. Lee CH, et al. Adenine Nucleotide Translocase 2 as an Enzyme Related to [(18)F] FDG Accumulation in Various Cancers. *Mol Imaging Biol.* 2019;21(4):722-30.
9. Dobin A, et al. STAR: ultrafast universal RNA-seq aligner. *Bioinformatics.* 2013;29(1):15-21.
10. Trapnell C, et al. Transcript assembly and quantification by RNA-Seq reveals unannotated transcripts and isoform switching during cell differentiation. *Nat Biotechnol.* 2010;28(5):511-5.
11. Trapnell C, et al. Differential analysis of gene regulation at transcript resolution with RNA-seq. *Nat Biotechnol.* 2013;31(1):46-53.
12. Anders S, et al. HTSeq--a Python framework to work with high-throughput sequencing data. *Bioinformatics.* 2015;31(2):166-9.
13. Love MI, et al. Moderated estimation of fold change and dispersion for RNA-seq data with DESeq2. *Genome Biol.* 2014;15(12):550.
14. von Mering C, et al. STRING: a database of predicted functional associations between proteins. *Nucleic Acids Res.* 2003;31(1):258-61.
15. Berridge BR, et al. Non-proliferative and Proliferative Lesions of the Cardiovascular System of the Rat and Mouse. *J Toxicol Pathol.* 2016;29(3 Suppl):1S-47S.
16. da Silva FS, et al. CmyoSize: An ImageJ macro for automated analysis of cardiomyocyte size in images of routine histology staining. *Ann Anat.* 2022;241:151892.
17. Linders AN, et al. Evaluation of Senescence and Its Prevention in Doxorubicin-Induced Cardiotoxicity Using Dynamic Engineered Heart Tissues. *JACC CardioOncol.* 2023;5(3):298-315.

Supplemental Figures

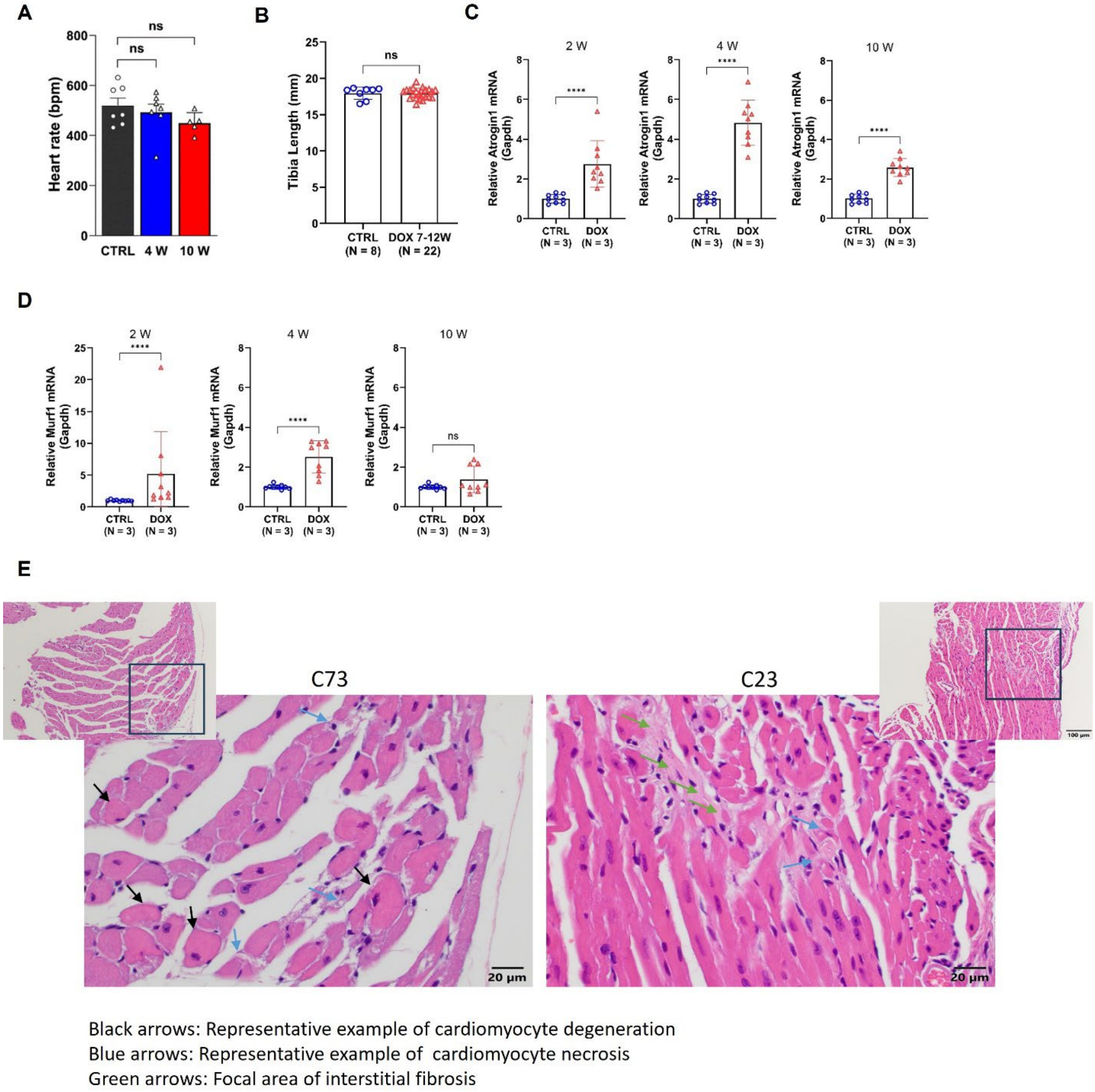

Supplemental Figure 1. ***Systemic DOX administration induces cardiomyocyte atrophy detectable by echo before the onset of fibrosis.*** **(A)** Comparison of heart rate from the left ventricle (LV) serial echo. CTRL and DOX groups (control, n=7; 4 weeks, n=6; and 10 weeks, n=5) were used for analysis. **(B)** Comparison of TL in CTRL and DOX-treated mice at the time of sacrifice. TL was measured ex vivo by digital calipers. **(C)** *Mafbx*/atrogin1 mRNA expression was determined by RT-qPCR at 2, 4, and 10 W using three different primers (n = 3 mice/group). The *Gapdh* gene was used as a reference gene in cardiac tissue. **(D)** *Murf1* mRNA was determined by RT-qPCR at 2, 4, and 10 W using three different primers (n = 3 mice/group). The primers are listed

in Supplemental Table 6. **(E)** Representative examples of histopathological analysis identifying focal areas of cardiomyocyte degeneration and necrosis or interstitial fibrosis. C73 and C23 are identification numbers given to mice receiving DOX. Data are presented as the mean ± s.d. Statistical comparisons were performed using a one-way ANOVA (**A**) or a two-tailed Mann-Whitney test (**B-D**). * $p < 0.05$; *** $p < 0.001$; **** $p < 0.0001$. DOX = doxorubicin; echo = echocardiogram; CTRL = control; BW = body weight; TL = tibia length; W = weeks.

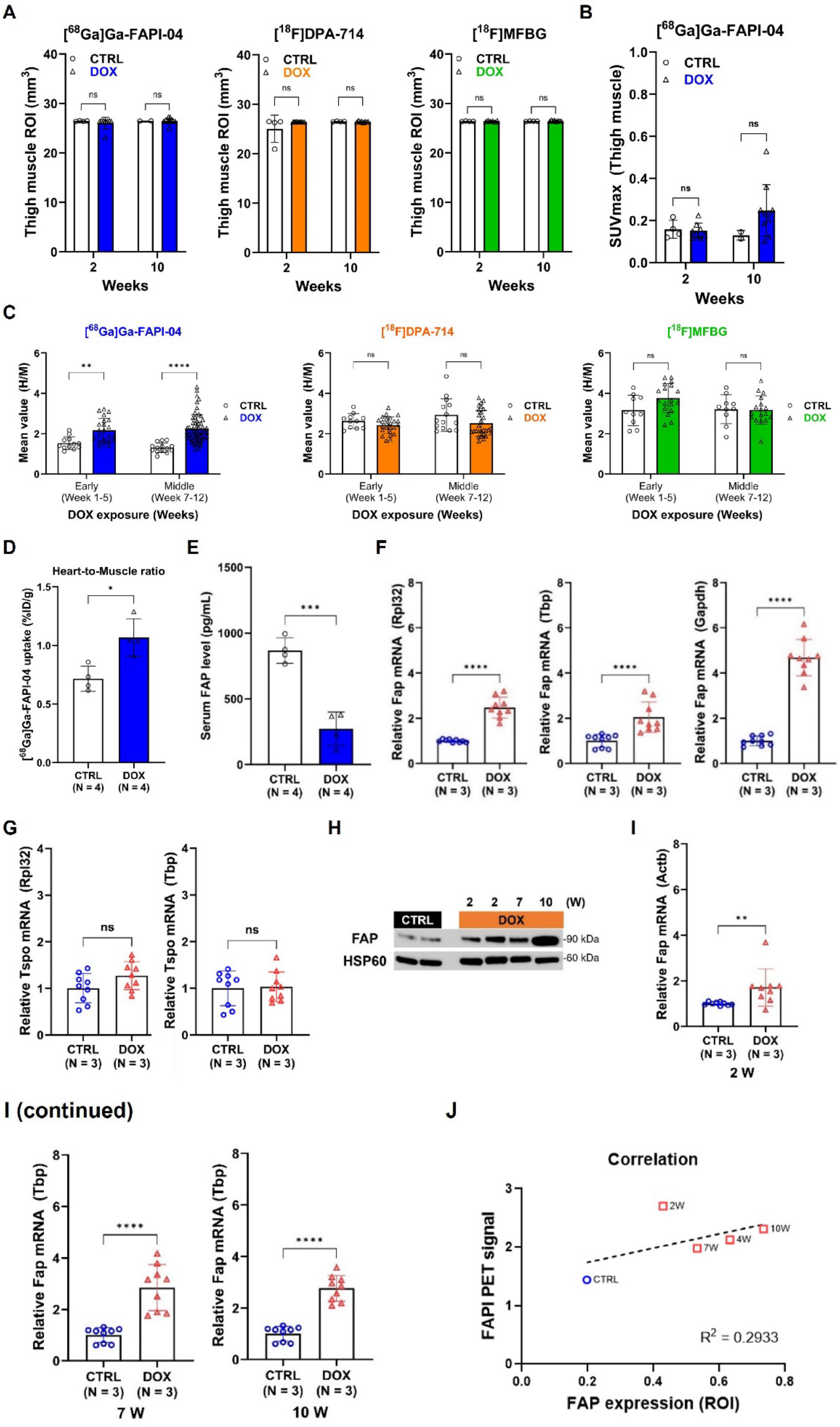
A
[68Ga]Ga-FAPI-04
[18F]DPA-714
[18F]MFBG
Thigh muscle ROI (mm3)
CTRL
DOX
ns
Weeks
B
[68Ga]Ga-FAPI-04
SUVmax (Thigh muscle)
C
Mean value (H/M)
Early (Week 1-5)
Middle (Week 7-12)
DOX exposure (Weeks)
D
Heart-to-Muscle ratio
[68Ga]Ga-FAPI-04 uptake (%ID/g)
CTRL (N = 4)
DOX (N = 4)
E
Serum FAP level (pg/mL)
F
Relative Fap mRNA (Rpl32)
Relative Fap mRNA (Tbp)
Relative Fap mRNA (Gapdh)
CTRL (N = 3)
DOX (N = 3)
G
Relative Tspo mRNA (Rpl32)
Relative Tspo mRNA (Tbp)
H
CTRL
DOX
2 2 7 10 (W)
FAP
HSP60
-90 kDa
-60 kDa
I
Relative Fap mRNA (Actb)
2 W
I (continued)
7 W
10 W
J
Correlation
FAPI PET signal
FAP expression (ROI)
CTRL
2W
4W
7W
10W
R2 = 0.2933

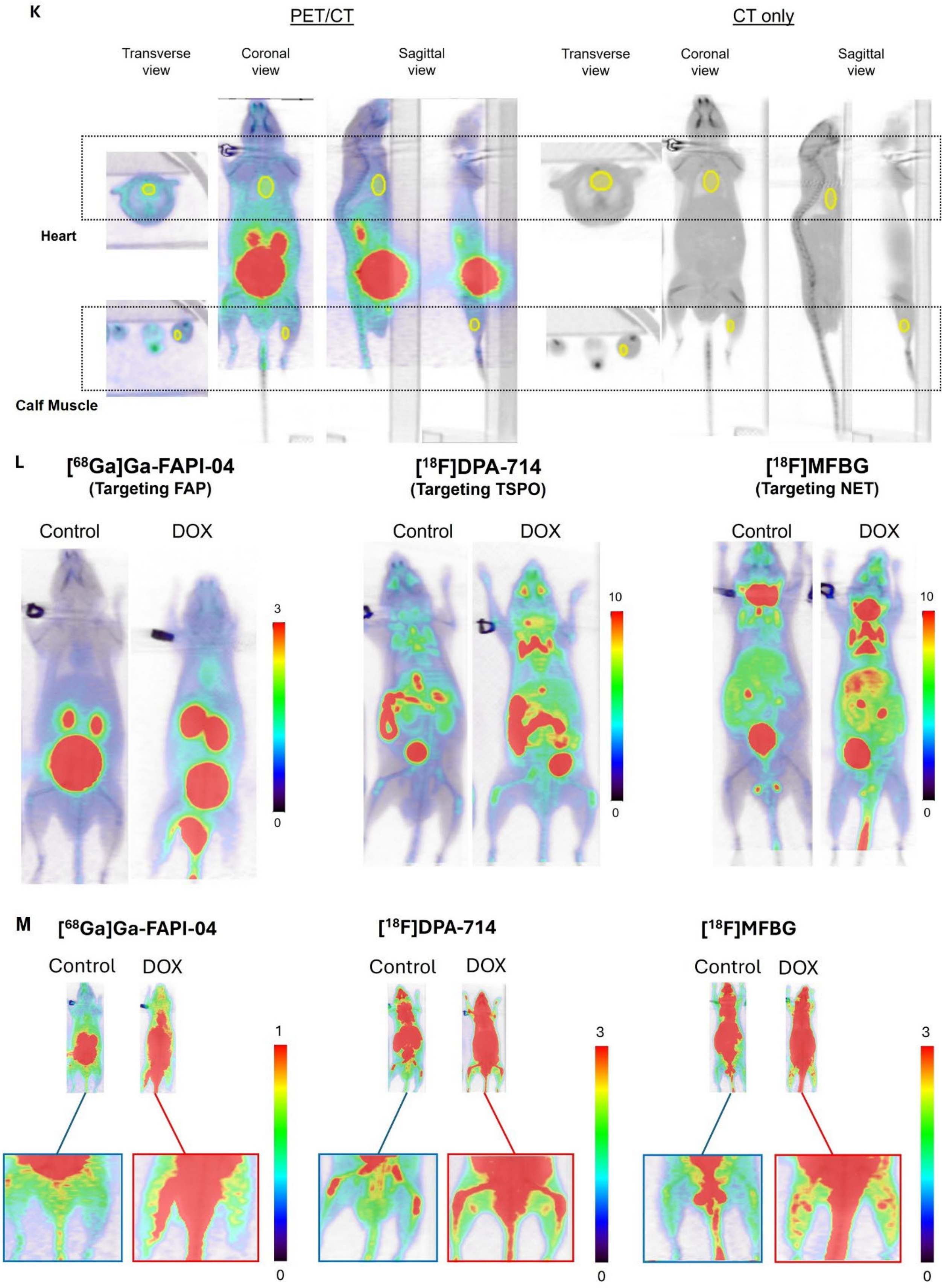
K
PET/CT
CT only
Transverse view
Coronal view
Sagittal view
Transverse view
Coronal view
Sagittal view
Heart
Calf Muscle
L
[68Ga]Ga-FAPI-04
(Targeting FAP)
[18F]DPA-714
(Targeting TSPO)
[18F]MFBG
(Targeting NET)
Control
DOX
Control
DOX
Control
DOX
3
0
10
0
10
0
M
[68Ga]Ga-FAPI-04
[18F]DPA-714
[18F]MFBG
Control
DOX
Control
DOX
Control
DOX
1
0
3
0
3
0

Supplemental Figure 2. ***Early-stage FAP expression is detectable with [^{68}Ga]Ga-FAPI-04 PET*.** **(A)** Comparison of the volume of interest (VOI) over the calf muscle between the CTRL and DOX groups. No significant difference was observed between animals and groups. **(B)** SUVmax of [^{68}Ga]Ga-FAPI-04 in the calf muscle at 2 and 10 weeks. **(C)** Reconstruction of image-derived cardiac uptake of [^{68}Ga]Ga-FAPI-04, [^{18}F]DPA-714, and [^{18}F]MFBG from Figure 2D based on early (week 1-5) or middle (week 7-12) phase. **(D)** Cardiac uptake of [^{68}Ga]Ga-FAPI-04 determined by ex vivo biodistribution. The activity in the heart tissue was normalized by the activity in the calf muscle. **(E)** Serum FAP concentration was determined by ELISA. **(F)** RT-qPCR quantification of cardiac *Fap* mRNA expression at 4 weeks using *Rpl32*, *Tbp*, and *Gapdh* as reference genes (n = 3 mice/group) to minimize the tissue heterogeneity. **(G)** RT-qPCR quantification of cardiac *Tspo* mRNA expression at 4 weeks using *Rpl32* and *Tbp* as reference genes (n = 3 mice/group) to minimize the tissue heterogeneity. **(H)** FAP protein expression at the corresponding qPCR time points as determined by Western blot. **(I)** RT-qPCR (n = 3 mice/group) was performed at 2, 7, and 10 weeks for the quantification of *Fap* mRNA expression using three different primers. *Actb* (2 weeks) and *Tbp* (7 and 10 weeks) were used as reference genes in cardiac tissue. The primers are listed in Supplemental Table 6. **(J)** Correlation between the averaged FAPI PET signal and the representative protein level of 2, 4, 7, and 10 weeks. (**K**) Representative VOIs drawn over the heart and the calf muscle. (**L**) Representative coronal maximum intensity projections (MIPs) for mice imaged at 2 weeks. Images were scaled to highlight differences in cardiac uptake. (**M**) Representative coronal MIPs scaled to highlight differences in calf muscle uptake in mice imaged at 2 weeks. Data are presented as the mean ± s.d. Statistical comparisons were performed using a 2-way ANOVA (**A**, **C**), an unpaired two-tailed t-test (**B**, **D**, and **E**), a two-tailed Mann-Whitney test (**F**, **G**, and **I**), or a Pearson correlation test (**J**). * $p < 0.05$; ** $p < 0.01$; **** $p < 0.0001$. AMIDE = A Medical Imaging Data Examiner (7).

A

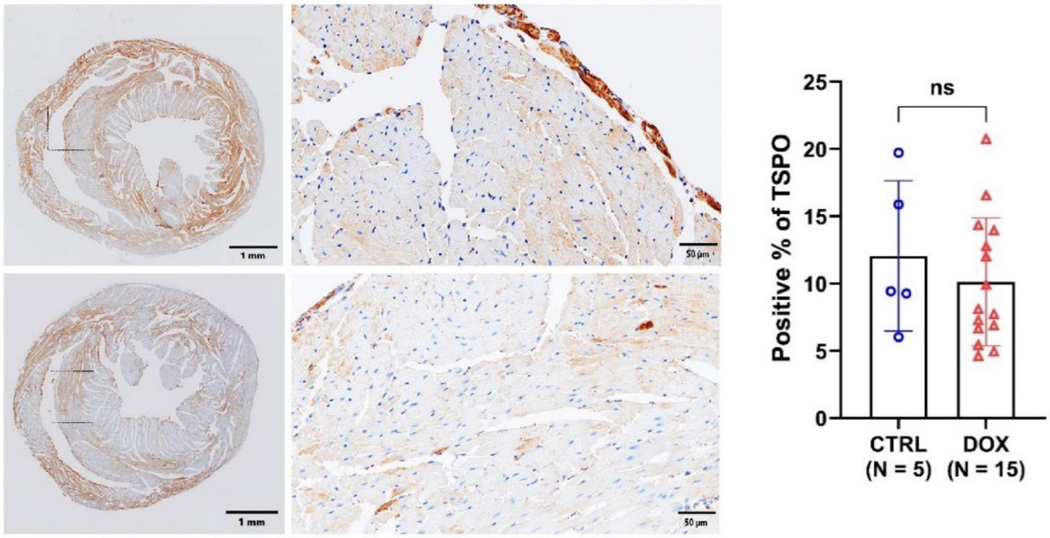

CTRL
DOX
1 mm
50 µm
ns
Positive % of TSPO
25
20
15
10
5
0
CTRL
(N = 5)
DOX
(N = 15)

B

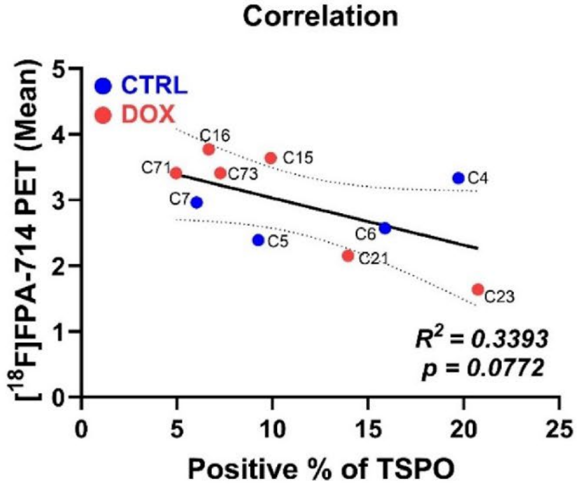

Correlation
CTRL
DOX
[18F]FPA-714 PET (Mean)
C16
C15
C71
C73
C4
C7
C6
C5
C21
C23
R2 = 0.3393
p = 0.0772
Positive % of TSPO
0
5
10
15
20
25

C

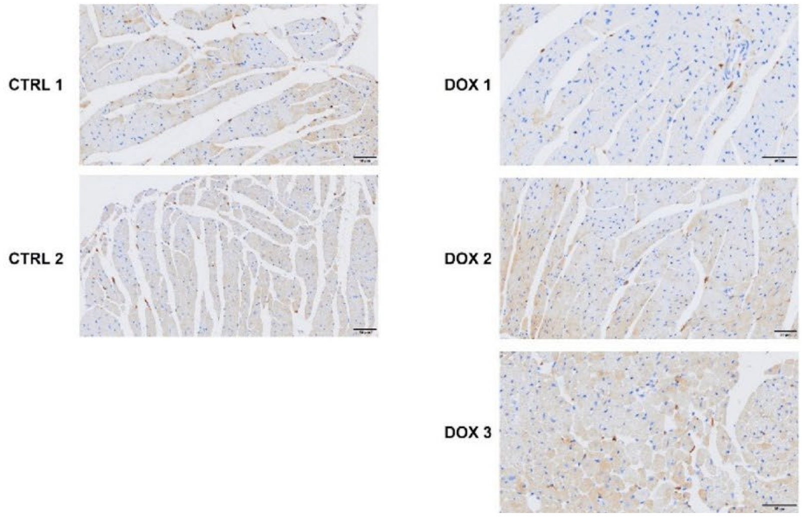

CTRL 1
CTRL 2
DOX 1
DOX 2
DOX 3

Supplemental Figure 3. ***[^{18}F]DPA-714 uptake is not significantly associated with the molecular and histopathological changes after DOX-induced cardiotoxicity.*** **(A)** Immunohistochemical staining of representative cardiac tissue slices. Slices were stained with anti-TSPO antibody. Positive regions were quantitated using QuPath software. Scale bar: 1 mm and 50 µm. **(B)** There was no significant correlation between [^{18}F]DPA-714 PET signal and TSPO expression ($p = 0.0772$). **(C)** Sectioned heart tissue was stained with recombinant anti-CD11b antibody. Representative CTRL samples (n = 2) and DOX samples (n = 3). Scale bar: 50 µm. Statistical comparisons were performed using an unpaired two-tailed t-test (**A**) or a Pearson correlation test (**B**).

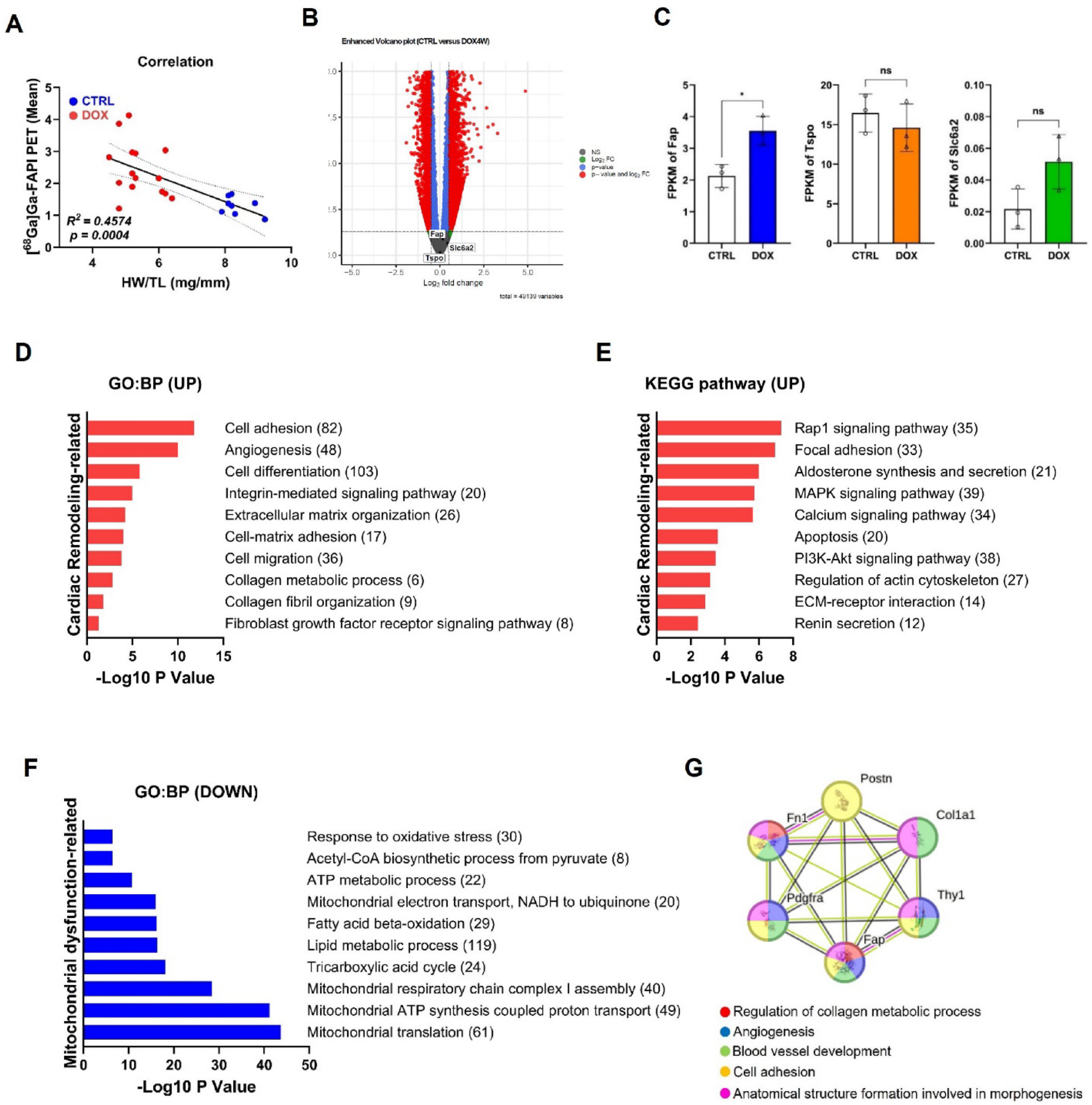

Supplemental Figure 4. ***DOX triggers early activation of genes linked to cardiac remodeling and mitochondrial dysfunction.*** **(A)** Correlation between HW/TL ratio and [^{68}Ga]Ga-FAPI-04 PET signal from cardiac tissues (*p = 0.0004*). **(B)** EnhancedVolcano plot for *Fap*, *Tspo*, and *Slc6a2* from the bulk RNA sequencing at 4 weeks (Log2 fold change (FC) > |0.5|, P < 5e-2, y-axis limit < 10). **(C)** Comparison of *Fap*, *Tspo*, and *Slc6A2* expression levels after normalization by fragments per kilobase of transcript per million mapped fragments (FPKM). **(D)** Significantly upregulated GO:BPs involving tissue remodeling at 4 weeks. **(E)** Significantly upregulated KEGG pathways supporting tissue remodeling at 4 weeks. **(F)** Significantly downregulated GO:BPs involving michondrial dysfunction at 4 weeks. Parentheses of (D-F) indicate the number of identified genes of each GO:BP and KEGG pathway. **(G)** STRING database from six genes including Fap and their associated GO:BPs. Data are presented as the mean ± s.d. Statistical comparisons were made using a Pearson correlation test (**A**) or an unpaired two-tail t-test (**C**). * *p*

< *0.05*. GO:BP = gene ontology:biological process; KEGG = Kyoto Encyclopedia of Genes and Genomes; STRING = Search Tool for the Retrieval of Interacting Genes/Proteins.

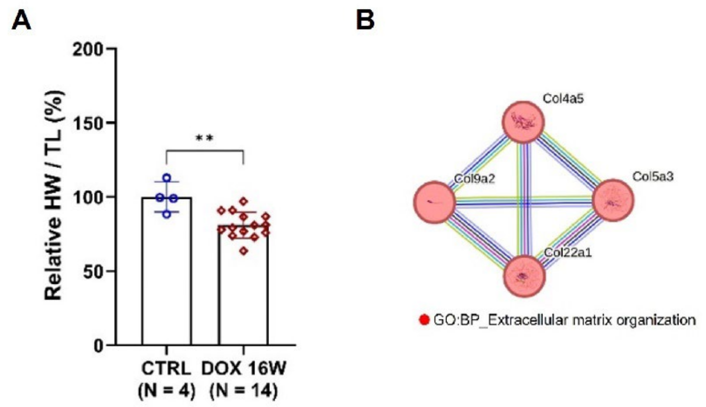

Supplemental Figure 5. ***Early FAPI PET predicts DOX-induced cardiac growth as a compensatory response to declining heart function.*** **(A)** HW/TL ratio between 16 weeks group. **(B)** STRING database for the top upregulated genes with enrichment scores at 16 weeks and their associated GO:BPs. Data are presented as the mean ± s.d. Statistical comparisons were performed using a two-tailed Mann-Whitney test. ** $p < 0.01$.

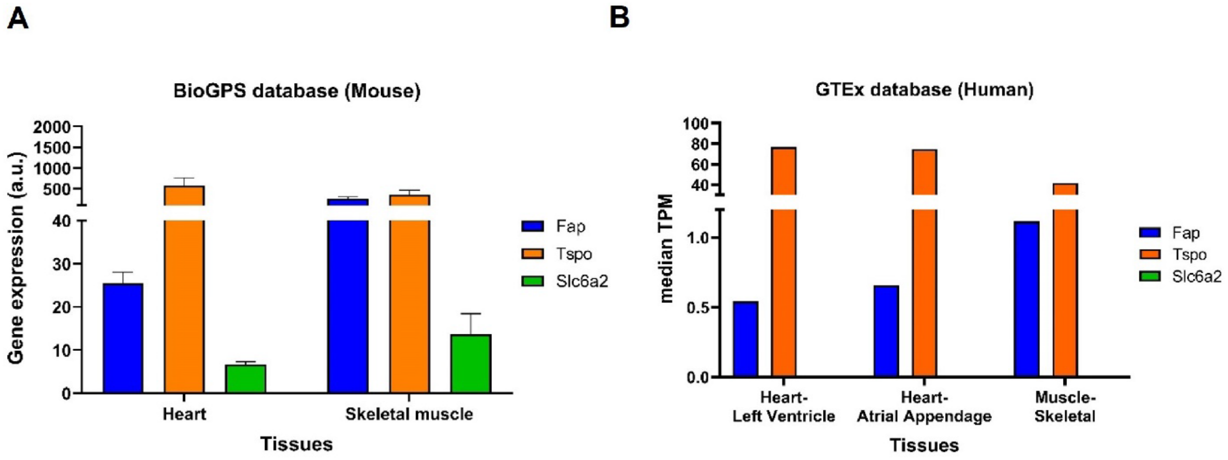

Supplemental Figure 6. ***Baseline expression levels of Fap, Tspo, and Slc6a2 in the heart and skeletal muscle, as derived from gene databases, and their correlation with [^{68}Ga]Ga-FAPI-04 PET imaging.*** **(A)** Gene expression profiles of *Fap*, *Tspo*, and *Slc6a2* in relevant mouse tissues were obtained from the BioGPS database. **(B)** *FAP*, *TSPO*, and *SLC6A2* transcript levels in human tissues were obtained from the Genotype-Tissue Expression project (GTEx). The supplemental Table 13 displayed the numbers in detail.

SUPPLEMENTAL TABLES

Supplemental Table 1. Body weights of control (CTRL) and doxorubicin (DOX) mice.

	**Control (CTRL)**		**Doxorubicin (DOX)**		**P values**
**Weeks (W)**	**Number (n)**	**AVG ± SD (g)**	**Number (n)**	**AVG ± SD (g)**	
**0**	18	26.6 ± 1.6	64	26.8 ± 1.7	$p = 0.7680$
**1**	8	25.9 ± 1.7	20	22.4 ± 2.0	$p = 0.0002$
**4**	10	27.1 ± 2.7	21	22.3 ± 1.2	$p < 0.0001$
**9**	8	30.3 ± 1.8	25	20.3 ± 2.5	$p < 0.0001$
**11**	4	29.8 ± 1.3	13	19.5 ± 2.2	$p < 0.0001$
**16**	4	30.0 ± 1.8	14	22.5 ± 2.2	$p < 0.0001$

Supplemental Table 2. Heart weight (HW)-to-tibia length (TL) ratio (mg/mm).

Group 1

	Control (CTRL)		Doxorubicin (DOX)		P values
**Weeks (W)**	**Number (n)**	**AVG ± SD**	**Number (n)**	**AVG ± SD**	
**7**	0		4	5.36 ± 0.85	
**9**	0		4	5.75 ± 0.61	
**10**	0		5	5.38 ± 0.76	
**11**	0		5	5.24 ± 0.53	
**12**	11	8.86 ± 0.92	4	4.79 ± 1.00	
**Total**	11	8.86 ± 0.92	22	5.30 ± 0.85	$p < 0.0001$

Group 2

	Control (CTRL)		Doxorubicin (DOX)		
**Weeks (W)**	**Number (n)**	**AVG ± SD**	**Number (n)**	**AVG ± SD**	
**16**	4	7.32 ± 0.73	14	5.93 ± 0.64	$p = 0.0046$

Supplemental Table 3. Summary of quantitative metrics of cardiac tissue.

Type	Mouse ID	Weeks (Tissue collected)	H&E morphometric analysis	FAPI-PET (mean)	FAP ISH (H-score)	Collagen %	TSPO-PET (mean)	TSPO %
CTRL	C4	12	X	1.38	1.76	8.41	3.33	19.72
	C5	12		1.67	1.25	-	2.39	9.26
	C6	12	X	1.18	0.97	5.70	2.57	15.89
	C7	12		1.11	0.54	-	2.96	6.02
	C8	12	X	1.04	-	7.23	2.54	9.44
DOX	C71	7		1.43	1.10	-	3.41	4.96
	C73	7	X	1.27	0.73	-	3.41	7.27
	C88	7		-	5.41	-	3.07	7.74
	C95	7		-	8.15	-	3.88	5.45
	C15	9	X	3.04	3.40	4.97	3.64	9.90
	C16	9	X	2.16	2.51	6.81	3.77	6.67
	C21	9	X	1.85	1.56	8.93	2.15	13.95
	C23	9	X	2.54	3.19	5.79	1.64	20.76
	C35	9		-	-	5.22	-	16.56
	C37	9		-	-	6.45	-	12.01
	C38	9		-	-	5.59	-	-
	C40	10		-	-	6.10	-	-
	C47	10		-	-	5.67	-	4.62
	C30	11	X	3.87	2.97	6.92	-	12.77
	C32	11	X	2.08	1.09	6.26	-	8.11
	C33	11		2.41	1.79	4.97	-	6.90
	C36	11		-	-	9.70	-	-
	C46	12		-	-	5.98	2.41	14.34
	C48	12		-	-	7.14	-	-

X indicates that the sample was used for the indicated analysis.

Supplemental Table 4. Summary of mice imaged by [^{68}Ga]Ga-FAPI-04, [^{18}F]DPA-714, or [^{18}F]MFBG PET.

	**PET probes**	**[^{68}Ga]Ga-FAPI-04**		**[^{18}F]DPA-714**		**[^{18}F]MFBG**	
	**Weeks**	**Control (n)**	**DOX (n)**	**Control (n)**	**DOX (n)**	**Control (n)**	**DOX (n)**
**Early Phase**	**1**	4	8	4	8	3	3
	**2**	4	8	4	8	4	6
	**4**	4	6	3	8	3	9
**Middle phase**	**7**	4	16	4	8	3	5
	**9**	4	22	4	6	0	0
	**10**	6	10	4	8	4	8
	**11**	0	10	0	0	0	0
	**12**	0	0	2	6	3	4
**Late phase**	**16**	4	4	0	0	0	0

Supplemental Table 5. Image-based quantification of heart-to-muscle ratio in mice imaged by [^{68}Ga]Ga-FAPI-04, [^{18}F]DPA-714, or [^{18}F]MFBG at 2 or 10 weeks after initial exposure to DOX.

PET probes	Weeks	Groups	Heart ROI (H)	Calf Muscle ROI (M)	H/M ratio
[^{68}Ga]Ga-FAPI-04	2	CTRL	0.257	0.197	1.307
			0.307	0.232	1.323
			0.295	0.196	1.509
			0.603	0.278	2.171
		DOX	1.078	0.438	2.458
			0.948	0.359	2.639
			0.524	0.240	2.182
			0.632	0.235	2.685
			1.376	0.437	3.150
			1.638	0.704	2.327
			1.785	0.554	3.221
			1.412	0.480	2.939
[^{18}F]DPA-714	2	CTRL	4.588	1.633	2.810
			4.369	1.924	2.270
			4.748	1.574	3.016
			5.012	1.915	2.617
		DOX	5.381	1.982	2.715
			5.382	2.820	1.909
			5.393	2.348	2.297
			5.507	2.932	1.878
			8.344	2.844	2.934
			7.561	2.546	2.970
			7.328	2.751	2.664
			6.400	2.447	2.615
[^{18}F]MFBG	2	CTRL	3.362	1.132	2.971
			3.499	1.349	2.593
			4.836	1.299	3.724
			4.323	1.140	3.792
		DOX	7.935	1.660	4.779
			7.997	2.285	3.500
			5.704	1.806	3.159
			6.385	1.845	3.461
			7.159	1.745	4.102
			3.399	1.397	2.434
[^{68}Ga]Ga-FAPI-04	10	CTRL	0.585	0.360	1.623
			0.440	0.321	1.372
			0.062	0.037	1.689
			0.067	0.053	1.266
			0.061	0.052	1.171
			0.055	0.047	1.169
		DOX	0.615	0.300	2.048
			1.095	0.389	2.817
			1.035	0.451	2.296
			0.685	0.329	2.085

			0.785	0.326	2.409
			1.725	0.483	3.571
			0.998	0.612	1.631
			0.941	0.497	1.892
			2.317	1.146	2.023
			0.926	0.406	2.280
[18F]DPA-714	10	CTRL	1.839	0.852	2.158
			1.982	0.934	2.123
			1.819	0.803	2.266
			2.023	0.932	2.171
		DOX	3.580	1.906	1.878
			2.981	1.389	2.147
			3.826	2.204	1.736
			3.058	1.463	2.090
			3.009	1.423	2.115
			3.133	1.186	2.642
			3.537	1.793	1.972
			3.730	1.751	2.131
[18F]MFBG	10	CTRL	2.898	1.236	2.346
			2.852	0.815	3.499
			3.179	1.079	2.945
			3.440	0.991	3.471
		DOX	4.854	1.813	2.677
			6.193	1.927	3.215
			5.486	1.945	2.820
			4.630	1.542	3.001
			7.850	2.070	3.792
			6.993	2.110	3.314
			9.237	2.291	4.032
			4.502	2.803	1.606

Supplemental Table 6. Summary of target and reference mouse genes for RT-qPCR in CTRL and DOX heart tissues.

**Gene name**	**Gene Symbol**	**Sequences (5' to 3')**
Fibroblast activation protein alpha	Fap #1	Forward: CTC CCT CGT CCA ATT CAG TAT C
		Reverse: GTG GAT CTC CTG GTC TTT GTT
	Fap #2	Forward: CCA CGG AAC AGC AGA TGA TAA
		Reverse: TAC CAC ATC GCC TGG AAA TC
	Fap #3	Forward: CAC CTG ATC GGC AAT TTG TG
		Reverse: CCC ATT CTG AAG GTC GTA GAT GT
Translocator protein 18 kDa	Tspo #1	Forward: ACT GTA TTC AGC CAT GGG GTA
		Reverse: ACC ATA GCG TCC TCT GTG AAA
	Tspo #2	Forward: CTA GCT TGC AGA AAC CCT CTT
		Reverse: GTG AAA CCT CCC AGC TCT TT
	Tspo #3	Forward: TGC TGG TCT ACC TGG TCT TA
		Reverse: GGT ATA ACT GTG TCT GCA GGA G
Thy-1 cell surface antigen	Thy1 #1	Forward: GCT CTC AGT CTT GCA GGT GTC
		Reverse: CAG GCG AAG GTT TTG GTT CA
	Thy1 #2	Forward: TGC TCT CAG TCT TGC AGG TG
		Reverse: TGG ATG GAG TTA TCC TTG GTG TT
Periostin	Postn #1	Forward: TGG TAT CAA GGT GCT ATC TGC G
		Reverse: AAT GCC CAG CGT GCC ATA A
	Postn #2	Forward: GGG AGC CAC TAC CAC TCA G
		Reverse: GTA CGT GTA TGA CCC TTT TCC TT
Collagen type I alpha 1 chain	Col1a1 #1	Forward: GCT CCT CTT AGG GGC CAC T
		Reverse: ATT GGG GAC CCT TAG GCC AT
	Col1a1 #2	Forward: GCT CCT CTT AGG GGC CAC T
		Reverse: CCA CGT CTC ACC ATT GGG G
	Pdgfra #1	Forward: TCC ATG CTA GAC TCA GAA GTC A
		Reverse: TCC CGG TGG ACA CAA TTT TTC

Platelet-derived growth factor receptor alpha	Pdgfra #2	Forward:	ATG AGA GTG AGA TCG AAG GCA
		Reverse:	CGG CAA GGT ATG ATG GCA GAG
Fibronectin 1	Fn 1 #1	Forward:	ATG TGG ACC CCT CCT GAT AGT
		Reverse:	GCC CAG TGA TTT CAG CAA AGG
	Fn 1 #2	Forward:	GCT CAG CAA ATC GTG CAG C
		Reverse:	CTA GGT AGG TCC GTT CCC ACT
F-box protein 32 (Atrogin 1)	Fbxo32 #1	Forward:	GAG TGG GAG AGG TGT AGA AAT G
		Reverse:	CTC TGG CCA TGA CCT AAT ATG G
	Fbxo32 #2	Forward:	CAC TTC TTC CTT CAG TCG TAG AG
		Reverse:	CCA GGA GAT GGT GCT GAT AAA
	Fbxo32 #3	Forward:	GCG CCA TGG ATA CTG TAC TT
		Reverse:	ATC AGC TCC AAC AGC CTT AC
Tripartite motif containing 63 (MuRF 1)	Trim63 #1	Forward:	CAG AGG CAG TTG GAT CTC TAT G
		Reverse:	TGA GGC AGA GTC TCT CTA TGT
	Trim63 #2	Forward:	GAG TGG GTT TGG AGA CAA AGA
		Reverse:	TCC ATC AGG AAT CAG GCT AGA
	Trim63 #3	Forward:	GGA CTA CTT TAC TCT GGA CTT AGA AC
		Reverse:	CAG CCT CCT CTT CTG TAA ACT C
Ribosomal protein L32	Rpl32	Forward:	GCC TCT GGT GAA GCC CAA G
		Reverse:	TTG TTG CTC CCA TAA CCG ATG T
TATA box binding protein	Tbp	Forward:	TAT GAC CCC TAT CAC TCC TG
		Reverse:	TTC TTC ACT CTT GGC TCC TGT
Glyceraldehyde-3-phosphate dehydrogenase isoform 1 and 2	Gapdh	Forward:	CTC CCA CTC TTC CAC CTT CG
		Reverse:	GCC TCT CTT GCT CAG TGT CC
Beta-Actin	Actb	Forward:	TCA GCA AGC AGG AGT ACG ATG
		Reverse:	AAC AGT CCG CCT AGA AGC ACT T

Supplemental Table 6. GO:BPs analysis by upregulated genes from the bulk RNA sequencing between CTRL and DOX 4 weeks groups

GO:BP (UP)	Term	Count	PValue	Genes
GO:0007155	Cell adhesion	82	1.6E-12	ACHE, PCDHGB7, CNTNAP1, PCDHGB6, CSF3R, COL16A1, PCDHGB5, ITGB4, PCDHGB4, ITGB3, PCDHGB2, ITGA2B, MSLN, PTPRF, ICAM1, COMP, STAB1, BOC, CYP1B1, CCN2, CCN1, SCARF2, SCARF1, PCDHGA8, PCDHGA7, EGFL7, PCDHGA6, PCDHGA5, PCDHGA4, VWF, PCDHGA3, PCDHGA2, PCDHGA1, PCDHGA9, CDH11, COL4A3, PCDHGB1, PRKD2, ITGA5, PLCB1, EPHA2, ENG, NLGN2, SRC, NRXN1, LAMA3, AMIGO2, PXN, PCDH15, NID2, THBS3, DPP4, ADGRG1, FLRT2, ADAM23, CDH23, NCAM1, MUC4, VCAM1, PCDH9, NEGR1, PCDHGC5, PCDHGC4, FN1, BMX, SULF1, L1CAM, SORBS3, PCDHGA10, MFAP4, PCDHGA11, PCDHGA12, NFASC, FES, COL7A1, ZYX, SSPO, CNTN2, ESAM, FAT3, RADIL, SDK2
GO:0001525	Angiogenesis	48	9.9E-11	ACVRL1, FOXC1, ROBO4, NOTCH1, FLT1, NRXN1, FLT4, ITGA2B, NPR3, HIF3A, GATA2, SAT1, PIK3R6, RASIP1, ACTG1, DLL4, ADGRG1, RNF213, HEY1, SOX17, SOX18, ADGRA2, GJA5, CYP1B1, FLNA, CCN2, VAV3, EGFL7, JUN, NOS3, CEMIP2, FN1, PTK2, FMNL3, PTPRB, TAL1, PRKD2, ADAM8, PRKD1, PNPLA6, ITGA5, PLCD3, PLCD1, RAPGEF3, EPHA2, FGFR1, ENG
GO:0030154	Cell differentiation	103	1.6E-06	LGALS3, SOX17, SOX18, CCN2, JUNB, SOX5, SEMA6B, TLE3, PRKCH, RFX2, SOX13, BIN1, PRKD1, EPHA2, RAI14, MEG3, SEMA7A, NOTCH1, ARHGEF28, NOTCH4, ILDR2, GATA2, SPATA6, RASGRP4, SBNO2, ADGRG1, CFAP69, JAG2, ODF2, CCDC136, RARRES2, OSGIN1, NR2F2, EIF2AK4, L1CAM, SMAD6, PTK2, BMP6, GADD45G, NFATC4, SMAD7, AHI1, BMP3, BMP1, FES, ID1, TMEM119, ID3, SSPO, RADIL, DZIP1, FGFR3, PIWIL2, ROBO4, FLT1, BNC1, TGFB1I1, TCF23, PIK3CD, SYNE1, ROBO1, C77080, NHSL2, CSRP2, CCHCR1, JAK3, UTP14B, UNC45A, EGFL7, APAF1, NRG1, TYK2, DUSP6, CIT, TAL1, ELF4, CDCP3, RAPGEF2, PPARG, IRF6, FOXC2, FOXC1, CATSPER2, MYRF, SRC,

				SEMA3D, USH1C, AMBRA1, MALT1, DLL4, MAPK7, ATOH8, FLNA, SLIT3, FLNB, STX2, TCF7L1, NOS3, KLF4, TTLL7, SMOC1, TLL1, AGRN
GO:0007229	Integrin-mediated signaling pathway	20	1.0E-05	VAV3, SEMA7A, ITGB4, SRC, ITGB3, LAMA3, PXN, ITGA2B, FN1, ADAM33, PTK2, ADAMTS20, ADAMTS14, ADAMTS19, ADAM4, ZYX, ADAM8, CCN2, ITGA5, LIMS2
GO:0030198	Extracellular matrix organization	26	6.2E-05	COL16A1, COL11A2, ADAMTS10, MMP23, ADAMTSL1, LGALS3, ADAMTS14, ADAMTS19, ADAMTS1, SPOCK2, ADAMTSL2, NPHP3, SLC39A8, CCN1, ATP7A, ADAMTS8, CSGALNACT1, COL27A1, FN1, PTK2, ADAMTS20, SMOC1, COL4A4, COL7A1, COL4A3, COL4A6
GO:0007160	Cell-matrix adhesion	17	1.1E-04	ZFP469, SNED1, VCAM1, ITGB4, ITGB3, PXN, ITGA2B, FN1, MSLN, L1CAM, NID2, ZYX, CNTN2, ADAM8, CCN2, ITGA5, MUC4
GO:0016477	Cell migration	36	1.6E-04	FOXC1, FLT1, ITGB4, SRC, ITGB3, LAMA3, PXN, PDGFB, PIK3CD, IRS2, MST1R, IQGAP1, PIK3C2A, PTPRF, ADGRG1, KCNQ1OT1, CCN2, RHBDF1, VAV3, CARMIL1, NRG1, CDC42BPB, MERTK, L1CAM, PTK2, BCR, FMNL3, CCDC88A, FMNL1, ARC, FMNL2, CORO6, ELMO1, CORO7, EPHA2, ENG
GO:0032963	Collagen metabolic process	6	1.6E-03	SMPD3, P2RX7, PRKCD, ID1, P3H3, TNS2
GO:0030199	Collagen fibril organization	9	1.6E-02	ZFP469, COMP, FOXC2, ADAMTS14, FOXC1, COL27A1, COL11A2, CYP1B1, ATP7A
GO:0008543	Fibroblast growth factor receptor signaling pathway	8	5.2E-02	FLRT2, CHRD, PDGFB, IQGAP1, CCN2, FGFR3, FGFR1, TBX2

Supplemental Table 7. KEGG pathways by upregulated genes from the bulk RNA sequencing between CTRL and DOX 4 weeks groups

**KEGG_PATHWAY (UP)**	**Term**	**Count**	**PValue**	**Genes**
mmu04015	Rap1 signaling pathway	35	4.8E-08	FLT1, SRC, ITGB3, FLT4, ITGA2B, PDGFB, ADCY4, PIK3CD, ADCY1, RASGRP2, SIPA1L3, ADCY7, ACTG1, SIPA1L1, AKT3, SCARF2, PRKCG, VAV3, ARAP3, MAPK12, GNAO1, MAPK11, PLCB3, PLCB4, ID1, RAPGEF2, PRKD2, PRKD1, RAPGEF5, PLCB1, FGFR3, SIPA1, RAPGEF3, EPHA2, FGFR1
mmu04510	Focal adhesion	33	1.2E-07	SHC2, FLT1, ITGB4, SRC, ITGB3, FLT4, LAMA3, ITGA2B, PXN, PDGFB, PIK3CD, THBS3, MYLK, ACTG1, COMP, ERBB2, AKT3, FLNA, FLNB, PRKCG, VAV3, JUN, VWF, ACTN1, FN1, ACTN4, PTK2, COL4A4, COL4A3, COL4A6, ZYX, ITGA5, PPP1R12C
mmu04925	Aldosterone synthesis and secretion	21	1.0E-06	PRKCG, NPR1, ADCY4, ITPR1, ITPR2, CACNA1D, ATP2B2, ITPR3, ADCY1, ADCY7, NR4A1, PLCB3, PLCB4, STAR, NPPA, PRKD2, PRKD1, PLCB1, SCARF2, CAMK1G, CREB5
mmu04010	MAPK signaling pathway	39	1.9E-06	FLT1, RASGRF2, FLT4, PDGFB, CACNA1A, CACNA1D, ARRB2, RASGRP2, CACNA1E, RASGRP4, IKBKB, MAPK7, ERBB2, AKT3, FLNA, FLNB, MAP3K8, MAP3K6, PRKCG, MAP4K2, JUN, DUSP6, MAPK12, GADD45G, CDC25B, NR4A1, MAPK11, DDIT3, RASA2, TAOK2, FAS, RAPGEF2, MAP3K14, FGFR3, HSPA1B, EPHA2, HSPA1A, MAP3K12, FGFR1
mmu04020	Calcium signaling pathway	34	2.3E-06	PTGFR, CHRM3, FLT1, FLT4, ATP2A3, PDGFB, ADCY4, ITPR1, ITPR2, CACNA1A, CACNA1D, ITPR3, ADCY1, MST1R, CACNA1E, ADCY7, MYLK, ERBB2, SCARF2, PRKCG, NOS3, ATP2B2, GRIN2C, TPCN2, P2RX7, P2RX6, PLCB3, PLCB4, PLCD3, PLCB1, FGFR3, CAMK1G, PLCD1, FGFR1
mmu04210	Apoptosis	20	2.6E-04	DFFB, JUN, APAF1, ITPR1, TNFRSF10B, PIK3CD, ITPR2, ITPR3,

				PTPN13, ACTG1, GADD45G, IKBKB, CASP12, DDIT3, AKT3, FAS, RIPK1, MAP3K14, SPTAN1, BCL2L1
mmu04151	PI3K-Akt signaling pathway	38	3.6E-04	CRTC2, FLT1, CSF3R, ITGB4, PKN3, ITGB3, FLT4, LAMA3, ITGA2B, PDGFB, IL4RA, PIK3CD, PIK3R6, THBS3, IKBKB, COMP, GNG2, ERBB2, AKT3, JAK3, PCK2, VWF, NOS3, FN1, OSMR, PTK2, NR4A1, COL4A4, COL4A3, RPS6KB2, COL4A6, MTCP1, ITGA5, FGFR3, EPHA2, BCL2L1, FGFR1, CREB5
mmu04810	Regulation of actin cytoskeleton	27	7.3E-04	CHRM3, ITGB4, SRC, ARPC1B, ITGB3, ITGA2B, PXN, PDGFB, PIK3CD, IQGAP1, MYLK, ACTG1, C7, AKT3, VAV3, ACTN1, FN1, ACTN4, BAIAP2, SSH3, PTK2, RGCC, ARHGEF1, ITGA5, PPP1R12C, FGFR3, FGFR1
mmu04512	ECM-receptor interaction	14	1.4E-03	VWF, ITGB4, ITGB3, LAMA3, ITGA2B, FN1, THBS3, COMP, FRAS1, COL4A4, COL4A3, COL4A6, ITGA5, AGRN
mmu04924	Renin secretion	12	3.8E-03	GUCY1A1, PLCB3, PLCB4, NPR1, NPPA, ITPR1, ITPR2, CACNA1D, ITPR3, CLCA3A1, PLCB1, SCARF2

Supplemental Table 8. GO:BPs analysis by downregulated genes from the bulk RNA sequencing between CTRL and DOX 4 weeks groups

GO:BP (DOWN)	Term	Count	PValue	Genes
GO:0032543	Mitochondrial translation	61	2.0E-44	MRPS16, MRPS14, FASTKD2, MRPS11, MRPS12, MRPL39, MRPL36, MRPL37, MRPL34, MRPL35, MRPL32, MRPL41, MRPL4, MRPL42, MRPL2, MRPL40, MRPL1, MRPL9, CHCHD1, MRPS27, MRPS24, MRPS22, MRPS23, MRPL49, MRPS2, MRPS21, MRPL47, MRPL45, MRPS7, MRPL46, MRPL43, MRPL44, MRPS18C, MRPL50, MRPL51, NOA1, AURKAIP1, MRPS35, MRPL18, MRPL19, MRPS31, MRPL16, MRPL58, MRPL14, MRPL15, MRPS30, MRPL12, MRPL57, MRPL13, MRPL10, MRPL54, MRPL55, MRPL11, IARS2, PTCD3, NDUFA7, GATC, MRPL28, MRPL21, MRPL22, MRPL30
GO:0042776	Mitochondrial ATP synthesis coupled proton transport	49	5.67E-42	NDUFA11, NDUFA12, NDUFA10, ATP5C1, NDUFC2, SDHC, NDUFC1, SDHD, SDHA, SDHB, ATP5J2, NDUFS8, NDUFS7, NDUFS6, NDUFS5, NDUFS4, NDUFS3, NDUFS2, NDUFS1, NDUFB9, STOML2, NDUFB8, NDUFB6, NDUFB10, NDUFB11, NDUFB5, NDUFB4, ATP5A1, NDUFB3, ATP5K, ATP5J, NDUFB1, ATP5H, ATP5O, ATP5B, ATP5E, ATP5D, NDUFV3, NDUFV2, NDUFV1, NDUFA9, NDUFA8, NDUFA7, NDUFA6, NDUFA5, ATP5PB, NDUFA3, NDUFA1, NDUFAB1
GO:0032981	Mitochondrial respiratory chain complex I assembly	40	3.29E-29	NDUFB9, NDUFB8, DMAC1, NDUFA11, NDUFB10, NDUFB6, NDUFB11, NDUFA12, NDUFB5, NDUFB4, NDUFA10, NDUFB3, NDUFB1, AIFM1, NDUFA9, NDUFA8, NDUFA6, NDUFA5, NDUFA3, NDUFA1, NDUFC2, NDUFC1, TIMM21, BCS1L, OXA1L, NDUFS8, NDUFS7, NDUFAF6, NDUFS6, NDUFS5, NDUFAF4, NDUFS4, NDUFAB1, TMEM126A, NDUFS3, NDUFAF3, NDUFS2, NDUFS1, NDUFAF1, TMEM126B
GO:0006099	Tricarboxylic acid cycle	24	7.9E-19	PDHA1, MDH1, MDH2, MRPS36, IDH3G, IDH1, IDH2, DLST, SDHC, PDHB, SDHD, SDHA, SDHB, FH1, CS, SUCLA2, NDUFS4, OGDH, IDH3B, SUCLG2, SUCLG1, ACO2, DLAT, IDH3A

GO:0006629	Lipid metabolic process	119	5.5E-17	LPGAT1, ACAA2, ECI1, HDLBP, ACSM5, COMT, LCLAT1, LACTB, AKR7A5, SCP2, MLYCD, HADH, PTGDS, ACAD11, ACOT7, DGAT2, ACSL1, OXSM, ECH1, ACOT13, INPP4B, CYP2U1, ACOT2, ACOT1, PLBD1, HACD1, RETSAT, INSIG2, MGST3, LPL, PLA2G5, ABHD5, ADIPOR1, ACAT1, ATP5B, PSAP, THEM4, ST3GAL5, RDH14, COX10, FDFT1, DECR1, GSTM2, EPHX2, ADHFE1, CIDEA, DOLK, CRLS1, ACADSB, PRDX6, ACSF2, CYP51, PM20D1, NCEH1, ECHDC3, DEGS1, NFE2L1, ACADVL, RAB7, TECR, VLDLR, AKR1B3, PIP4P2, CPT2, ACADL, SMPD1, B3GALT2, ACADM, ACADS, PLA2G12A, LYPLA1, PCYT1A, HMGCS1, GPX4, BCKDHB, PLAAT1, PLAAT3, PTGR2, HADHB, HADHA, PCCA, BDH1, ACOX1, ZADH2, CNEP1R1, EHHADH, B4GALT6, BCAT2, PAFAH1B1, PLIN5, ST6GALNAC6, PRKAA2, ECHS1, ATP5A1, FITM1, HSD17B4, FITM2, HSD17B7, HSD17B10, AGPAT2, AGPAT3, OXCT1, SC5D, GPAT3, SPTSSA, ACSS1, LDLR, CBR4, SLC16A1, PNPLA8, PTGES2, FAH, GPAM, LPCAT3, NDUFAB1, CHPT1, LPIN1, CRAT, PNPLA2
GO:0006635	Fatty acid beta-oxidation	29	6.6E-17	GCDH, ACADVL, ABCD3, ECHS1, ACAA2, ECI1, ECI2, HSD17B4, HSD17B10, MTLN, ACAT1, CPT2, SCP2, ACADM, HADH, ACAD11, DECR1, ACAD12, ECH1, PEX2, ACADSB, HADHB, PEX7, HADHA, ACOX1, EHHADH, ECHDC1, CRAT, TUBA8
GO:0006120	Mitochondrial electron transport, NADH to ubiquinone	20	8.99E-17	NDUFB9, NDUFA8, NDUFB8, NDUFA7, NDUFB6, BDNF, NDUFA10, NDUFC2, COQ9, DNAJC15, NDUFS8, NDUFS7, NDUFS6, NDUFS3, NDUFS2, NDUFS1, NDUFAF1, NDUFV2, DLD, NDUFV1
GO:0046034	ATP metabolic process	22	1.6E-11	ATP5PB, ENTPD5, ATP5A1, AK1, OLA1, ATP5K, ATP5C1, ATP5J, AK3, ATP1A2, AK4, ATP5H, ATP5O, ATP1B1, ATP5G1, CLPX, ATP5J2, ATP5B, GUK1, ATP5E, ATP5D, NDUFS1
GO:0006086	Acetyl-CoA biosynthetic process from pyruvate	8	3.46E-07	PDHX, PDHA1, MPC1, MPC2, VDAC1, PDHB, DLAT, DLD
GO:0006979	Response to oxidative stress	30	3.59E-07	NDUFA12, NDUFB4, ETFDH, LIAS, TXN2, PRDX3, PRDX2, PRDX5, AIFM1, PRDX1, BANF1, HMOX2, PRNP, NQO1, NDUFA6, GPX4, 1600014C10RIK, IDH1, SOD2,

				PRDX6, COQ7, SOD1, PINK1, NDUFS8, RCAN2, CAT, PPIF, MSRB2, NDUFS2

Supplemental Table 9. GO:BPs analysis through the STRING database from six-target genes that were highly expressed at 4 weeks by RT-qPCR

#Term ID	Term description	Strength	False Discovery Rate (FDR)	Matching proteins (Interesting targets)
GO:0010712	Regulation of collagen metabolic process	2.11	0.0467	Fn1,Fap
GO:0001525	Angiogenesis	1.62	0.0014	Fn1,Fap,Thy1,Pdgfra
GO:0001568	Blood vessel development	1.51	0.0011	Col1a1,Fn1,Fap,Thy1,Pdgfra
GO:0007155	Cell adhesion	1.3	0.0012	Fn1,Postn,Fap,Thy1,Pdgfra
GO:0048646	Anatomical structure formation involved in morphogenesis	1.25	0.0015	Col1a1,Fn1,Fap,Thy1,Pdgfra

Supplemental Table 10. Histologic examination of trichrome stain between 4 and 16 weeks in DOX groups.

Cardiac tissues	Week 4	Week 16
1	Normal	Fine interstitial collagen in left and right ventricular walls, multifocal
2	Normal	Normal
3	Normal	Normal
4	Normal	Interstitial collagen around vessel in left ventricle, focal
5	Normal	Normal
6	Normal	Fine interstital collagen in left ventricular wall, focal

Supplemental Table 11. The upregulated genes with the highest enrichment score from the DAVID database (2.87) at 16 weeks. The top GO:BPs of these genes indicated Extracellular matrix organization (P = 4.5e-04 through the STRING database)

Col4a5	collagen, type IV, alpha 5(Col4a5)
Col22a1	collagen, type XXII, alpha 1(Col22a1)
Col9a2	collagen, type IX, alpha 2(Col9a2)
Col5a3	collagen, type V, alpha 3(Col5a3)

Supplemental Table 12. The top seven genes with the highest log2FC values from DEGs at 16 weeks

**Positive top 7 genes**		
Gene name	Function	Reference
Sprr1a	Left ventricle (LV) SPRR1A was upregulated in patients with Heart failure with reduced ejection fraction (HFrEF)	(1)
Myh7	Increased MYH7 levels contribute to hypertrophic cardiomyopathy (HCM) and suggest a risk of abnormal cardiac function and heart failure	(2)
Adamtsl2	Elevated ADAMTSL2 expression in the heart, primarily by cardiac fibroblasts, is linked to heart failure	(3)
Slitrk4	SLITRK4 shows potential as a diagnostic biomarker for hypertrophic cardiomyopathy	(4)
Mthfd2	Elevated MTHFD2 expression in the heart is associated with cardiovascular diseases, potentially promoting cell proliferation and contributing to disease progression	(5)
Tgfb2	Increased TGFB2 expression in the heart reflects elevated TGF-β2 levels, a key protein in cardiac remodeling and fibrosis. Its overexpression can drive excessive extracellular matrix deposition, commonly linked to heart failure or myocardial infarction, and often occurs in response to cardiac injury or stress	(6)
Nmrk2	Elevated NMRK2 expression in the heart is a marker of stress or pathology, often associated with heart failure	(7)
**Negative top 7 genes**		
Acr	Low ACR expression is linked to heart failure, as it may exacerbate myocardial damage by impairing autophagy	(8)
Osgin1	Reduced OSGIN1 expression in the heart may signal a compromised ability to manage oxidative stress, potentially resulting in increased cell damage and contributing to the development of cardiovascular diseases.	(9)
Tmem150c	Reduced TMEM150c expression in the heart may impair blood pressure regulation, potentially leading to hypertension, tachycardia, and abnormal heart rate fluctuations	(10)
Scn4b	A decrease in SCN4B expression results in lower production of the beta-4 subunit, disrupting sodium channel function and potentially causing abnormal electrical signals in the heart.	(11)
Id1	Reduced ID1 expression in the heart is linked to impaired cardiac development, potentially causing structural defects such as abnormal trabeculation and vascularization issues	(12)
Ngf	Reduced NGF expression in the heart, leading to lower Nerve Growth Factor levels, is associated with conditions like congestive heart failure (CHF). This decrease can impair the function of sympathetic nerves, contributing to weakened cardiac function and further heart muscle damage	(13)
Gpcpd1	Reduced GPCPD1 expression in the heart may disrupt normal metabolic function, particularly in phospholipid metabolism, potentially affecting cardiac contractility and contributing to heart disease.	(14)

Supplemental Table 13. Bulk tissue gene expression in humans for Fap (ENSG00000078098.15), Tspo (ENSG00000100300.18), and Slc6a2 (ENSG00000103546.19). The data source was determined by GTEx Analysis Release V10 (dbGaP Accession phs000424.v10.p2) (TPM; Transcripts Per Million).

	FAP (median TPM)	TSPO (median TPM)	NET (median TPM)
Heart-Left Ventricle (n = 452)	0.5406	76.50	0.000
Heart - Atrial Appendage (n = 461)	0.6569	74.40	0.000
Muscle - Skeletal (n = 818)	1.114	41.62	0.007890
Cells-Cultured fibroblasts (n = 652)	103.8	503.0	0.006065

Supplemental Table 14. Summary of target and housekeeping proteins for western blot.

Antibody name	Abbreviation	Dilution	Catalog #	Company
❖ Primary				
Topoisomerase 2β	TOP2β	1:1000	20549-1-AP	Proteintech, USA
MAFbx (F-box protein 32)	Atrogin 1	1:500	sc-166806	Santa Cruz, USA
Muscle specific RING finger protein	MuRF1	1:500	sc-398608	Santa Cruz, USA
Fibroblast activation protein alpha	FAP	1:500	ab53066	Abcam, UK
PBR (Translocator protein 18 kDa)	TSPO	1:6000	ab109497	Abcam, UK
SLC6A2 (Norepinephrine Transporter)	NET	1:250	MBS540046	MyBioSource, USA
alpha Actinin	ACTN1	1:1000	ab68194	Abcam, UK
Transcription factor EB	TFEB	1:1000	A303-673A	ThermoFisher, USA
Matrix metalloproteinase 2 (MMP2)	MMP2	1:1000	87809	Cell Signaling Technology, USA
TIMP metallopeptidase inhibitor 2	TIMP2	1:1000	5738	Cell Signaling Technology, USA
Actin alpha 2, smooth muscle	α-SMA	1:1000	ab124964	Abcam, UK
Vimentin	Vimentin	1:1000	5741	Cell Signaling Technology, USA
Actin alpha 1, skeletal muscle	ACTA1	1:500	17521-1-AP	Proteintech, USA
Myosin heavy chain 7	MYH7	1:500	sc-53090	Santa Cruz, USA
Bcl-2-like protein 4	BAX	1:1000	2772	Cell Signaling Technology, USA
Heat shock protein family D member 1	HSP60	1:1000	12165	Cell Signaling Technology, USA
Vinculin	Vinculin	1:1000	4650	Cell Signaling Technology, USA
❖ Secondary				
Anti-mouse IgG, HRP-linked Antibody	Anti-mouse	1:2000	7076	Cell Signaling Technology, USA
Anti-rabbit IgG, HRP-linked Antibody	Anti-rabbit	1:2000	7074	Cell Signaling Technology, USA

References

1. Aonuma T, et al. Cardiomyocyte microRNA-150 confers cardiac protection and directly represses proapoptotic small proline-rich protein 1A. *JCI Insight.* 2021;6(18).
2. Gacita AM, et al. Genetic Variation in Enhancers Modifies Cardiomyopathy Gene Expression and Progression. *Circulation.* 2021;143(13):1302-16.
3. Rypdal KB, et al. The extracellular matrix glycoprotein ADAMTSL2 is increased in heart failure and inhibits TGFbeta signalling in cardiac fibroblasts. *Sci Rep.* 2021;11(1):19757.
4. Zheng X, et al. Identification and verification of promising diagnostic biomarkers in patients with hypertrophic cardiomyopathy associate with immune cell infiltration characteristics. *Life Sci.* 2021;285:119956.
5. Hitzel J, et al. Oxidized phospholipids regulate amino acid metabolism through MTHFD2 to facilitate nucleotide release in endothelial cells. *Nat Commun.* 2018;9(1):2292.
6. Deng Z, et al. TGF-beta signaling in health, disease, and therapeutics. *Signal Transduct Target Ther.* 2024;9(1):61.
7. Tannous C, et al. NMRK2 Gene Is Upregulated in Dilated Cardiomyopathy and Required for Cardiac Function and NAD Levels during Aging. *Int J Mol Sci.* 2021;22(7).
8. Zhou LY, et al. The circular RNA ACR attenuates myocardial ischemia/reperfusion injury by suppressing autophagy via modulation of the Pink1/ FAM65B pathway. *Cell Death Differ.* 2019;26(7):1299-315.
9. Khoi CS, et al. Oxidative Stress-Induced Growth Inhibitor (OSGIN1), a Target of X-Box-Binding Protein 1, Protects Palmitic Acid-Induced Vascular Lipotoxicity through Maintaining Autophagy. *Biomedicines.* 2022;10(5).
10. Lu HJ, et al. Tentonin 3/TMEM150C senses blood pressure changes in the aortic arch. *J Clin Invest.* 2020;130(7):3671-83.
11. Campuzano O, et al. Genetics and cardiac channelopathies. *Genet Med.* 2010;12(5):260-7.
12. Cunningham TJ, et al. Id genes are essential for early heart formation. *Genes Dev.* 2017;31(13):1325-38.
13. Kaye DM, et al. Reduced myocardial nerve growth factor expression in human and experimental heart failure. *Circ Res.* 2000;86(7):E80-4.
14. Aisyah R, et al. GDE5/Gpcpd1 activity determines phosphatidylcholine composition in skeletal muscle and regulates contractile force in mice. *Commun Biol.* 2024;7(1):604.
