# Supplementary material for: FAP PET identifies earlycardiac molecular changesinduced by doxorubicin chemotherapy
Source: JCI Insight. 2025 Oct 23;10(23):e191058. doi: 10.1172/jci.insight.191058 (PMC12890471; doi:10.1172/jci.insight.191058)

Figure 2

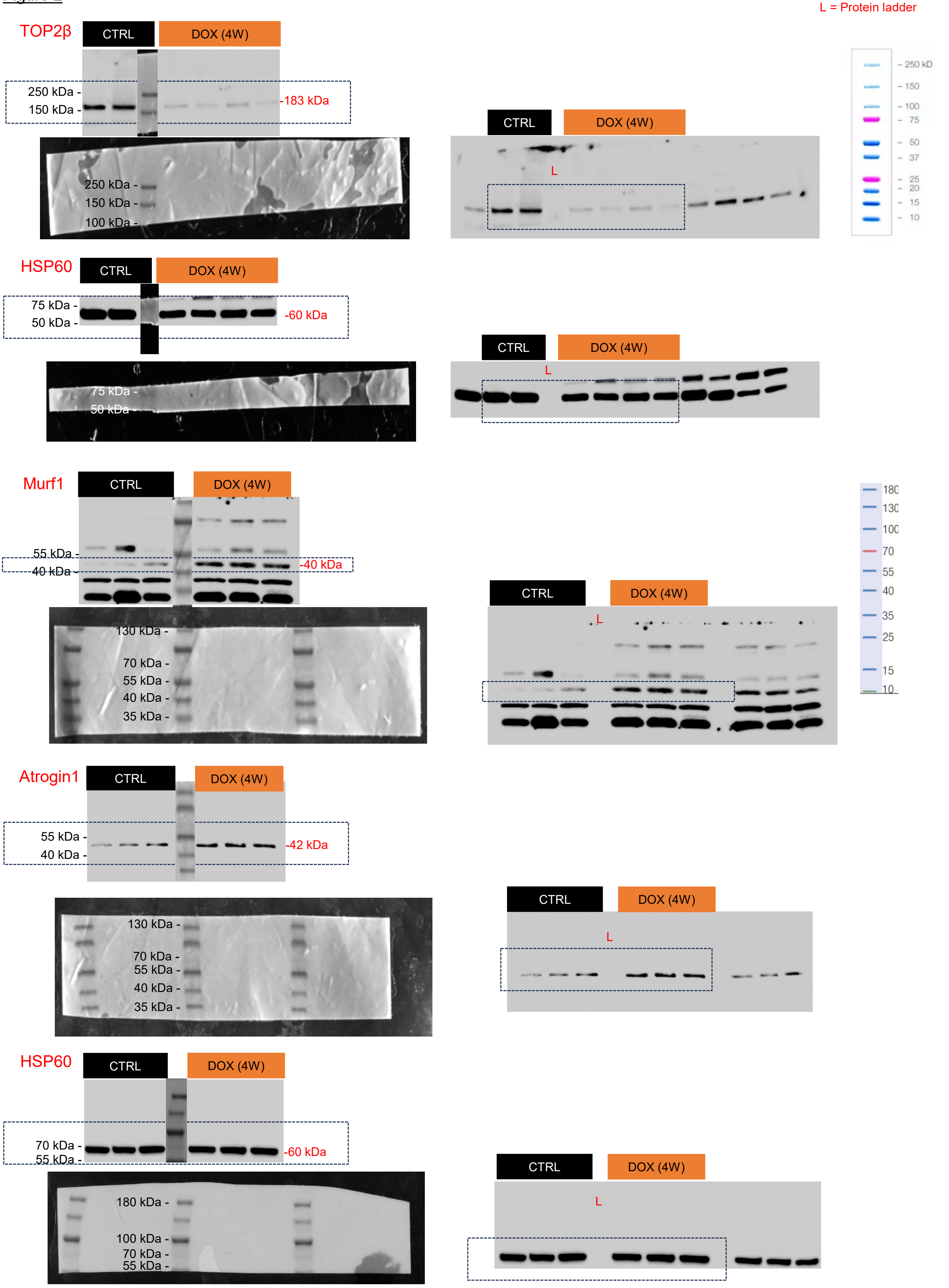
L = Protein ladder
TOP2β
CTRL
DOX (4W)
250 kDa -
150 kDa -
-183 kDa
250 kDa -
150 kDa -
100 kDa -
CTRL
DOX (4W)
L
- 250 kD
- 150
- 100
- 75
- 50
- 37
- 25
- 20
- 15
- 10
HSP60
CTRL
DOX (4W)
75 kDa -
50 kDa -
-60 kDa
75 kDa -
50 kDa -
CTRL
DOX (4W)
L
Murf1
CTRL
DOX (4W)
55 kDa -
40 kDa -
-40 kDa
130 kDa -
70 kDa -
55 kDa -
40 kDa -
35 kDa -
180
130
100
70
55
40
35
25
15
10
CTRL
DOX (4W)
L
Atrogin1
CTRL
DOX (4W)
55 kDa -
40 kDa -
-42 kDa
130 kDa -
70 kDa -
55 kDa -
40 kDa -
35 kDa -
CTRL
DOX (4W)
L
HSP60
CTRL
DOX (4W)
70 kDa -
55 kDa -
-60 kDa
180 kDa -
100 kDa -
70 kDa -
55 kDa -
CTRL
DOX (4W)
L

Figure 4

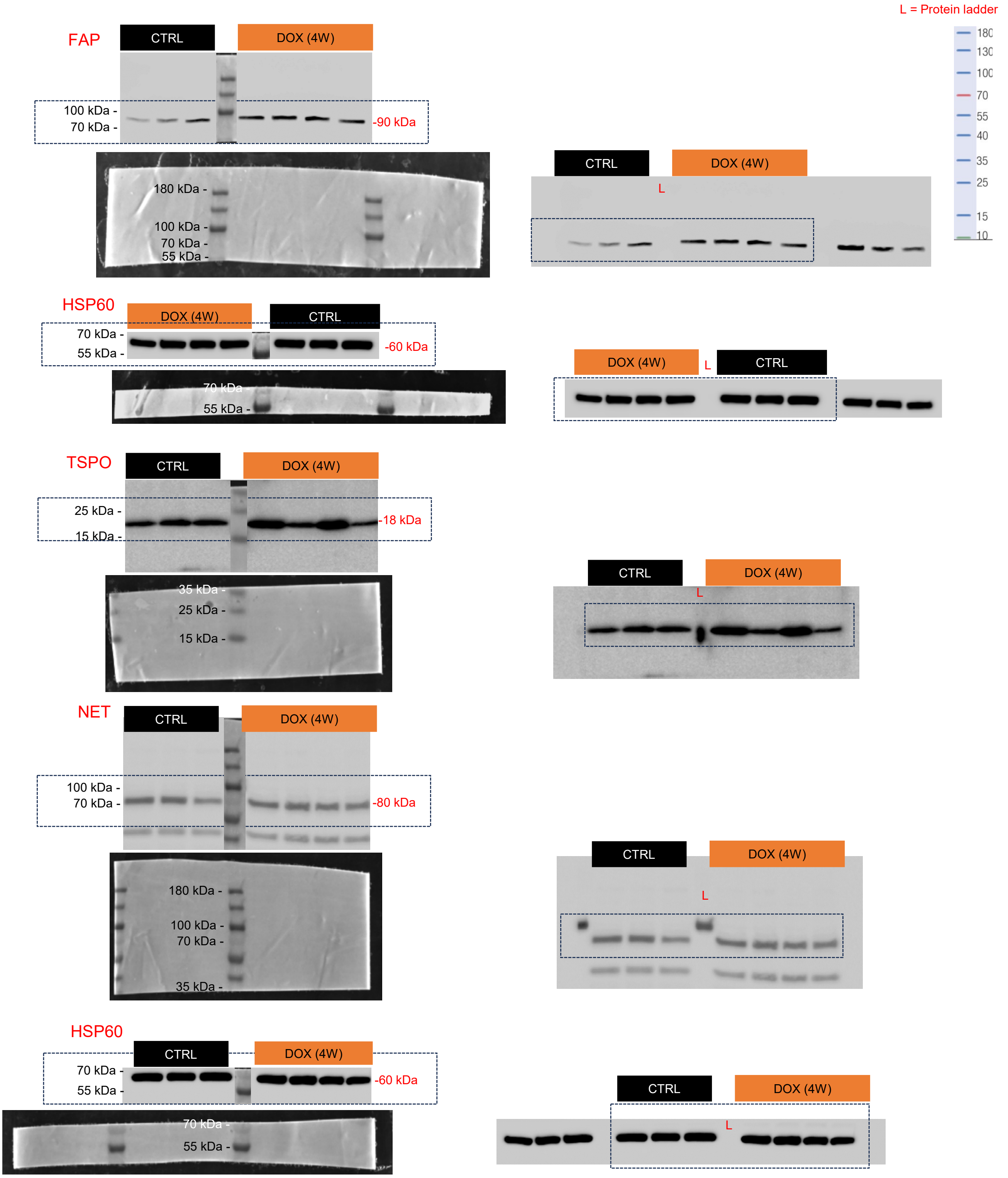

L = Protein ladder
FAP
CTRL
DOX (4W)
100 kDa -
70 kDa -
-90 kDa
180 kDa -
100 kDa -
70 kDa -
55 kDa -
L
180
130
100
70
55
40
35
25
15
10
HSP60
DOX (4W)
CTRL
70 kDa -
55 kDa -
-60 kDa
TSPO
25 kDa -
15 kDa -
-18 kDa
35 kDa -
NET
-80 kDa
35 kDa -

Figure 5

L = Protein ladder

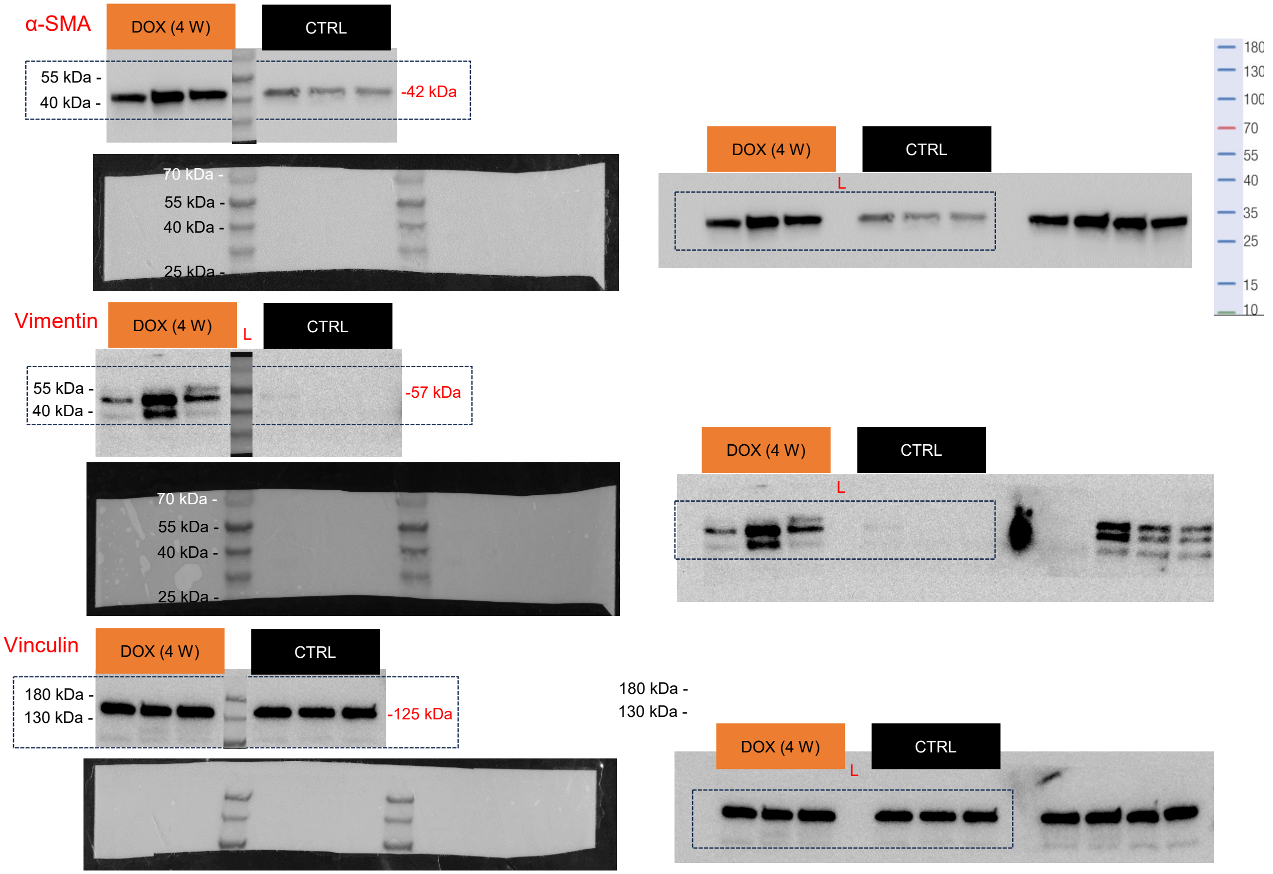

Figure 6

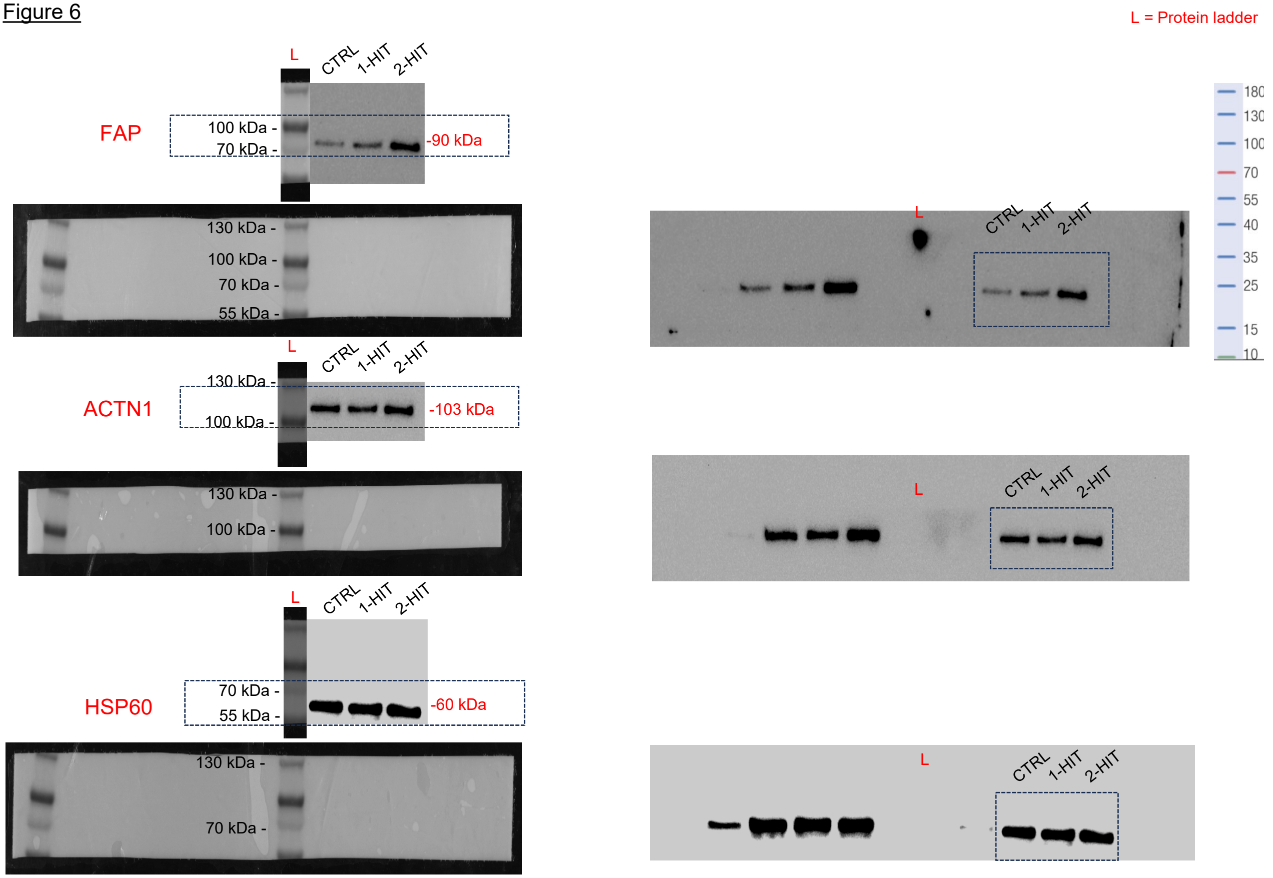
L = Protein ladder
L
CTRL
1-HIT
2-HIT
FAP
100 kDa -
70 kDa -
-90 kDa
130 kDa -
100 kDa -
70 kDa -
55 kDa -
ACTN1
130 kDa -
100 kDa -
-103 kDa
130 kDa -
100 kDa -
HSP60
70 kDa -
55 kDa -
-60 kDa
130 kDa -
70 kDa -
180
130
100
70
55
40
35
25
15
10

Figure 7

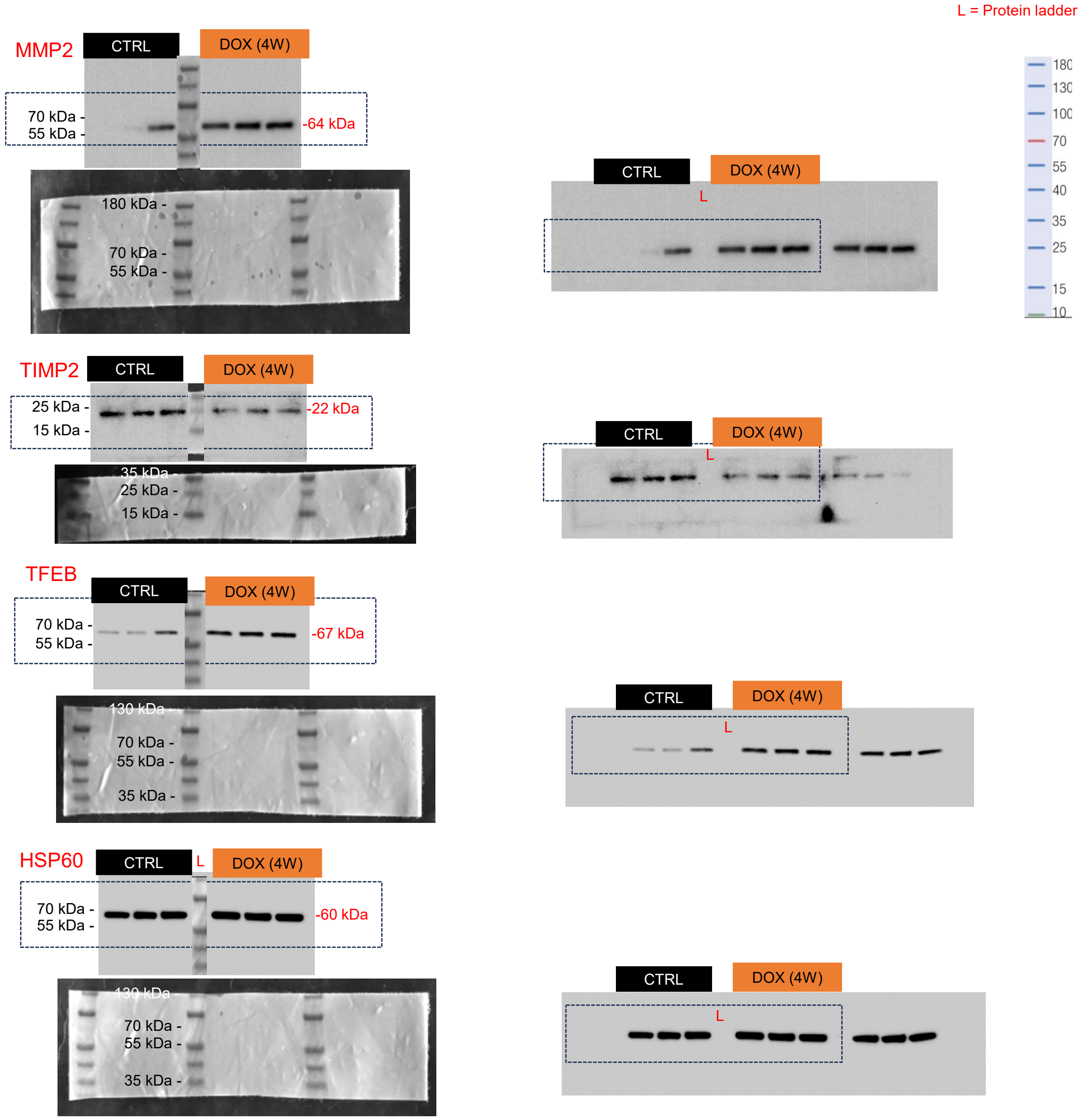
L = Protein ladder
MMP2
CTRL
DOX (4W)
70 kDa -
55 kDa -
-64 kDa
180 kDa -
70 kDa -
55 kDa -
L
TIMP2
25 kDa -
15 kDa -
-22 kDa
35 kDa -
25 kDa -
15 kDa -
TFEB
70 kDa -
55 kDa -
-67 kDa
130 kDa -
35 kDa -
HSP60
-60 kDa
180
130
100
70
55
40
35
25
15
10

Figure 8

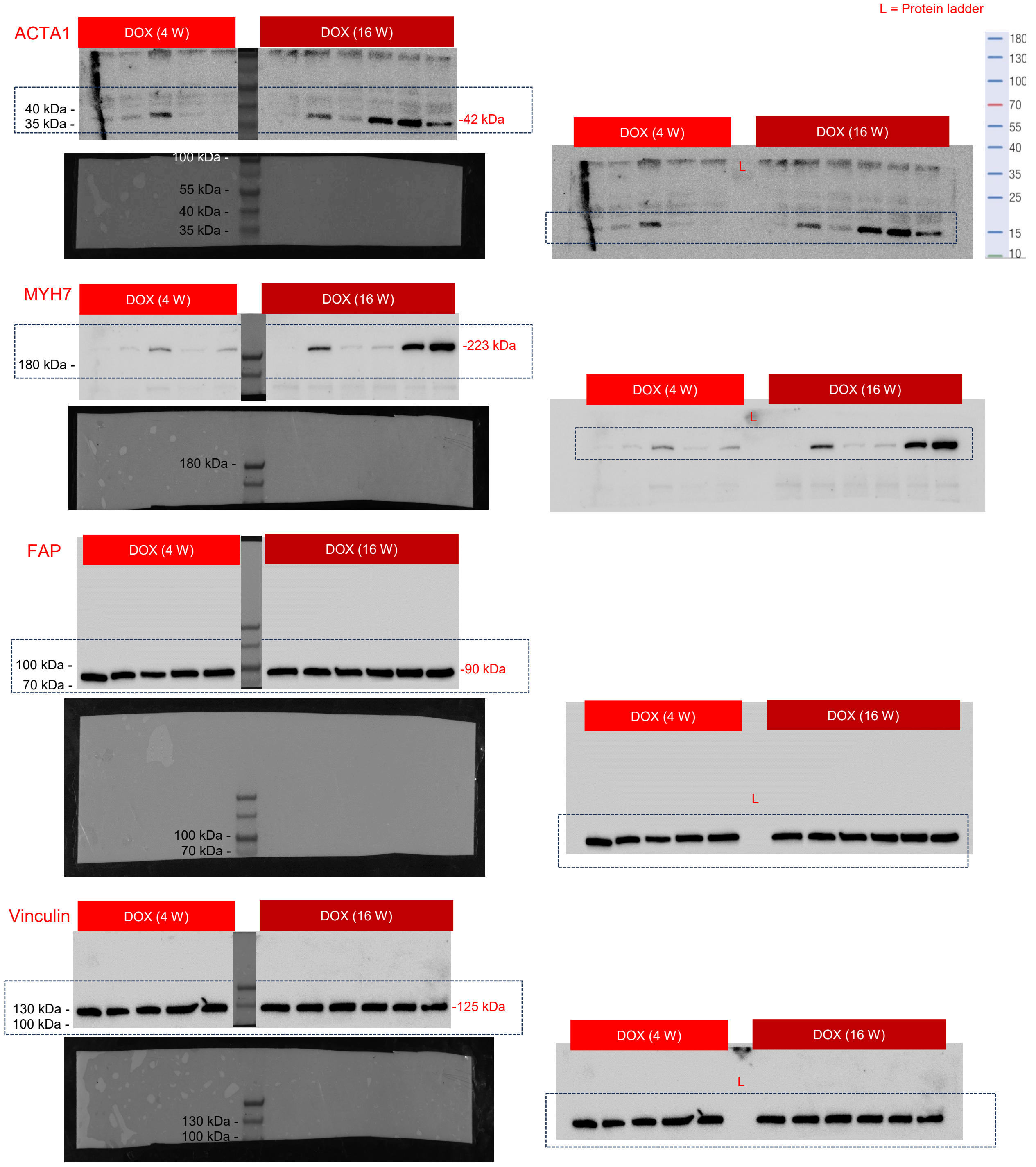
L = Protein ladder
ACTA1
DOX (4 W)
DOX (16 W)
40 kDa -
35 kDa -
-42 kDa
100 kDa -
55 kDa -
40 kDa -
35 kDa -
L
180
130
100
70
55
40
35
25
15
10
MYH7
180 kDa -
-223 kDa
FAP
100 kDa -
70 kDa -
-90 kDa
Vinculin
130 kDa -
100 kDa -
-125 kDa

Supplementary Figure 2

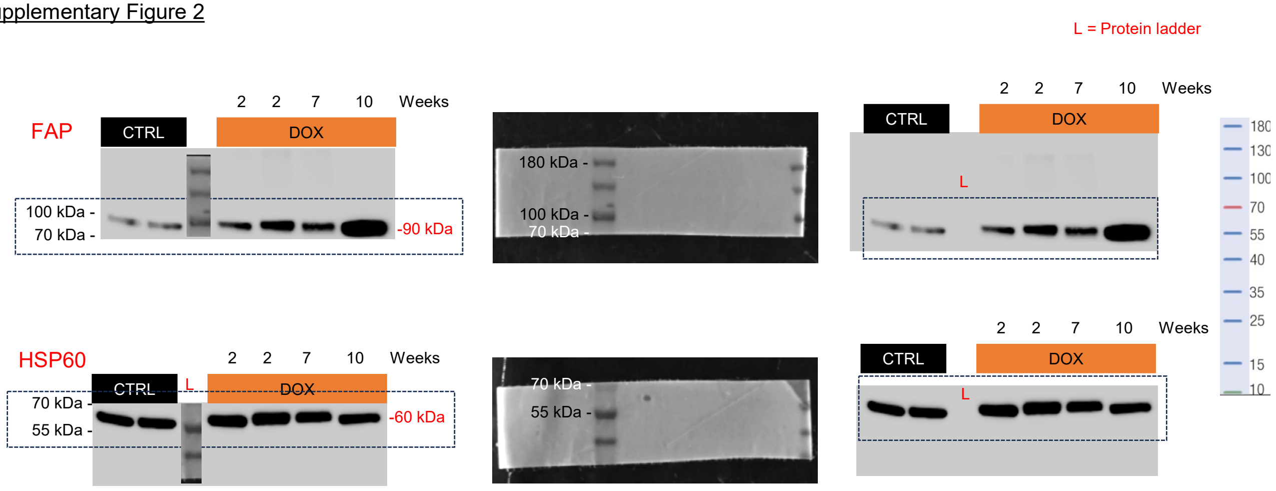

Supplement: Unedited blot and gel images [file jciinsight-10-191058-s038.pdf]
